# Genetic, phenotypic and ecological differentiation suggests incipient speciation in two *Charadrius* plovers along the Chinese coast

**DOI:** 10.1186/s12862-019-1449-5

**Published:** 2019-06-27

**Authors:** Xuejing Wang, Pinjia Que, Gerald Heckel, Junhua Hu, Xuecong Zhang, Chung-Yu Chiang, Nan Zhang, Qin Huang, Simin Liu, Jonathan Martinez, Emilio Pagani-Núñez, Caroline Dingle, Yu Yan Leung, Tamás Székely, Zhengwang Zhang, Yang Liu

**Affiliations:** 1 State Key Laboratory of Biocontrol, Department of Ecology, School of Life Sciences, Sun Yat-sen University, Guangzhou, 510275 China; 2 Ministry of Education Key Laboratory for Biodiversity and Ecological Engineering, College of Life Sciences, Beijing Normal University, Beijing, 100875 China; 3 Institute of Ecology and Evolution, University of Bern, Baltzerstrasse 6, 3012 Bern, Switzerland; 4 Chengdu Institute of Biology, Chinese Academy of Sciences, Chengdu, 610041 China; 5 Swiss Institute of Bioinformatics, Genopode, 1015 Lausanne, Switzerland; 6 Department of Environmental Science, Tunhai University, Taichun, Taiwan; 7 La Ferté Saint Aubin, France; 8 School of Biological Sciences, The University of Hong Kong, Hong Kong, SAR China; 9 Milner Center for Evolution, Department of Biology and Biochemistry, University of Bath, Bath, BA1 7AY UK

**Keywords:** Parapatry, Character displacement, Gene flow, Hybridization, Stable isotope analysis, Ecological niche

## Abstract

**Background:**

Speciation with gene flow is an alternative to the nascence of new taxa in strict allopatric separation. Indeed, many taxa have parapatric distributions at present. It is often unclear if these are secondary contacts, e.g. caused by past glaciation cycles or the manifestation of speciation with gene flow, which hampers our understanding of how different forces drive diversification. Here we studied genetic, phenotypic and ecological aspects of divergence in a pair of incipient shorebird species, the Kentish (*Charadrius alexandrinus*) and the White-faced Plovers (*C. dealbatus*), shorebirds with parapatric breeding ranges along the Chinese coast. We assessed divergence based on molecular markers with different modes of inheritance and quantified phenotypic and ecological divergence in aspects of morphometric, dietary and climatic niches.

**Results:**

Our integrative analyses revealed small to moderate levels of genetic and phenotypic distinctiveness with symmetric gene flow across the contact area at the Chinese coast. The two species diverged approximately half a million years ago in dynamic isolation with secondary contact occurring due to cycling sea level changes between the Eastern and Southern China Sea in the mid-late Pleistocene. We found evidence of character displacement and ecological niche differentiation between the two species, invoking the role of selection in facilitating divergence despite gene flow.

**Conclusion:**

These findings imply that ecology can indeed counter gene flow through divergent selection and thus contributes to incipient speciation in these plovers. Furthermore, our study highlights the importance of using integrative datasets to reveal the evolutionary history and assist the inference of mechanisms of speciation.

**Electronic supplementary material:**

The online version of this article (10.1186/s12862-019-1449-5) contains supplementary material, which is available to authorized users.

## Background

Understanding how strongly evolutionary processes (i.e. selection, gene flow and genetic drift) shape the divergence of closely related species has been a long-standing interest in evolutionary biology [1–5]. Allopatric speciation is conventionally considered to be the prevalent mode of speciation in which physical barriers completely restrict gene flow between two populations, facilitating the initiation of divergence through genetic drift or selection [6–8]. If populations remain isolated for a long enough period of time after divergence has been established, genome-wide differentiation is expected to be accumulated even with fixation of novel mutations. Such divergence can be maintained and eventually promote reproductive isolation due to genetic incompatibilities even in the presence of gene flow after secondary contact [9, 10].

An increasing number of studies have shown that divergence can arise and be maintained due to selection imposed by heterogeneous environments despite the homogenizing effect of gene flow [11, 12]. Under this scenario, divergent selection or ecologically-mediated sexual selection operating on certain traits can lead to reproductive isolation. Incompatibilities in a few “speciation genes [13, 14]” may be enough to constrain the homogenizing effect caused by gene flow, even at an early stage of speciation [15–17]. For instance, different host plant preferences result in divergence in digestive, physiological and morphological traits within sympatric ecotypes of *Timema* walking-stick insects [18]. In such a system, adaptive loci which are responsible for color pattern were confirmed to overcome local gene flow [19]. Locally-adapted phenotypes in body size are related to swimming and foraging ecology in guppies (*Poecilia reticulata*), which can maintain population diversification and fixed divergence in genomic regions [20] in the face of extensive gene flow [15]. Though evidence of speciation with gene flow is accumulating [11, 12, 21, 22], to what extent a balance between selection and gene flow can drive divergence remains largely unknown.

Confirming whether genetic differentiation between two taxa is due to strict allopatry or speciation-with-gene-flow remains challenging [23]. Towards this end, ideal study systems include populations found across a variety of environments, and which have a high propensity for introgressive hybridization such as in waterfowl (e.g. [24]) and shorebirds [25, 26]. Specifically, we expect differences in environments to confer differential selective regimes across the populations that would counteract the amalgamating forces of gene flow; and thus, providing a means to understand how selection, gene flow, and genetic drift effect the genomes of these species.

Shorebirds are a group of migratory species with remarkable movement ability. Seasonal migration may increase the probability of individual dispersal between populations [27, 28] and consequently drive frequent gene flow between geographically distant populations [29, 30]. Direct evidence, obtained using tracking approaches, confirmed that extremely long-distance gene flow can be mediated through individual movements among remote breeding colonies of pectoral sandpipers (*Calidris melanotos*) [31]. Range-wide phylogeographic studies also revealed extensive gene flow in several migratory shorebird species (e.g. [32–36]). This may result in weak genetic structure across a species and consequently prevent population-level divergence [25, 26].

**Fig. 1:**
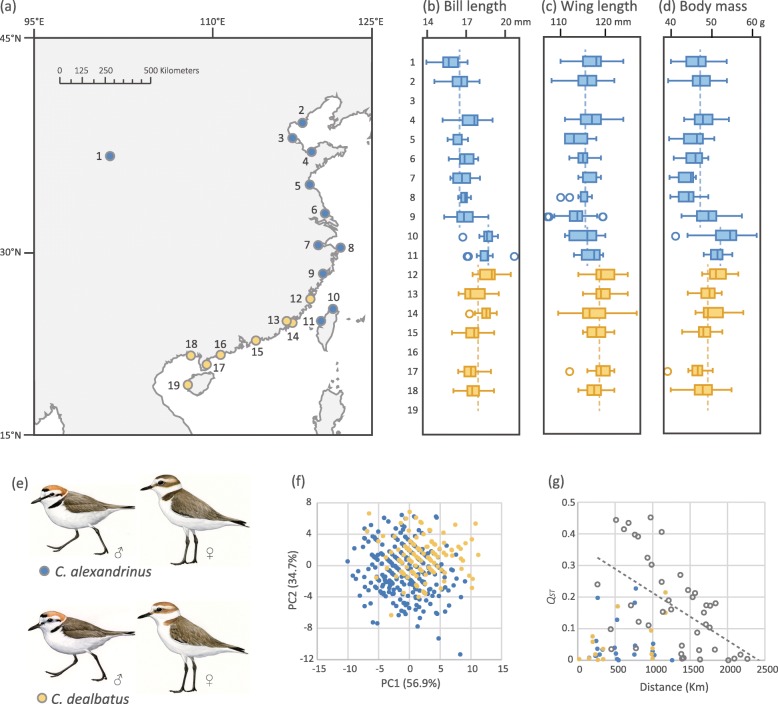
Sampling localities, morphology and trophic level differentiation of the Kentish Plover *Charadrius alexandrinus* (blue) and the White-faced Plover *C. dealbatus* (yellow). **a** Samples were from an inland site (1, Qinghai Lake), 16 Chinese mainland coastal localities (2, Tangshan; 3, Cangzhou; 4, Weifang; 5, Lianyungang; 6, Nantong; 7, Ningbo; 8, Zhoushan; 9, Wenzhou; 12, Fuzhou; 13, Xiamen; 14, Jinmen; 15, Shanwei; 16, Yangjiang; 17, Zhanjiang; 18, Beihai and 19, Dongfang), and two localities on Taiwan Island (10, Xinbei and 11, Zhanghua). Map source: National Geomatics Center of China (http://www.ngcc.cn). Differences observed between two *Charadrius* species in several characters. *C. alexandrinus* has on average shorter bill length (except for Taiwan populations) (**b**), wing length (**c**) and lower body mass (except for Taiwan populations) (**d**) than *C. dealbatus*. Localities with less than five measured adults were excluded in these analyses. **e** *C. alexandrinus* has darker plumage than *C. dealbatus* in general and males of the latter species have less black tinges on their face and neck during breeding season. Shaochong Peng drew the artwork of the two species of plovers and these images used with permission. **f** Plots of the first two principal components and their associated variance showed the subtle morphometric differences between the two species. **g** Pairwise morphological difference *Q*_*ST*_ plotted against geographical distance between coastal populations of *C. alexandrinus* and *C. dealbatus*. *Q*_*ST*_ values within species are marked by blue or yellow dots. *Q*_*ST*_ between species (grey circles) declined as the distance increased (regression showed as the dashed line)

In this study, we test the role of ecology and gene flow on the divergence of two shorebird species, the Kentish Plover (*Charadrius alexandrinus*) and the White-faced Plover (*C. dealbatus*; Fig. 1). *C. alexandrinus* breeds in coastal areas and inland lakes in Europe, Asia and North Africa [37]. A previous study uncovered low genetic differentiation of *C. alexandrinus* across the Eurasian continent, and also between continental and island populations in East Asia [32]. *C. dealbatus* was formerly described as a distinctive species [38, 39]. Breeding records of *C. dealbatus* were reported along the coast of China from Fujian to Hainan Island, and in south-central Vietnam [40], yet its geographic range and boundaries with *C. alexandrinus* are uncertain [41]. Previous studies have found subtle differences in morphometric, plumage and behavioral traits between the two taxa (Fig. 1d), leading to the erection of *C. dealbatus* as a full species [41, 42]. But no evidence of genetic differentiation suggested by the first genetic investigation, which concluded that these species may only differ in a few genomic regions [42]. Therefore, this taxonomic treatment has been accepted by some world bird checklists [43], but is yet to be accepted by others [44].

Here, we provide comprehensive data from multiple sources to explore divergence patterns between these two species of plovers. We carried out extensive sampling of breeding populations covering the potential area of contact along the Chinese coast. We then obtained genetic polymorphism data from multiple markers with various mutation rates, i.e. mitochondrial DNA, exons and autosomal microsatellites. Using these new datasets, it is possible to estimate the intensity and direction of gene flow [45, 46] as well as other important demographic parameters such as the effective population size (*N*e) and the timing of divergence [47] between *C. alexandrinus* and *C. dealbatus*. Because it is difficult to directly quantify divergent selection, it is necessary to make inference from the comparison of traits, which are potential targets of selection in order to offer hints on selective mechanisms in maintaining divergence [18, 48, 49]. By collecting data on morphology, diet, and environmental niche, we attempted to characterize geographic variation in genetic and phenotypic traits, estimate demographic history and intensity of gene flow, and infer the role of multiple factors, i.e. geographical premating isolation and gene flow during divergence, between these two taxa. Finally, because we applied multiple lines of evidence to demonstrate divergence patterns, we attempt to re-evaluate species delimitation between the two plovers. Taken together, our investigation provides new insights into the evolutionary history of two incipient bird species.

## Results

### Morphometric differentiation

Both plover species showed subtle but significant differences for most morphological traits (ANCOVA, *p* < 0.001, Fig. 1) even though there was considerable overlap in morphology between the species at the level of the individual (Fig. 1f). On average, *C. dealbatus* had a longer bill (17.91 ± 0.94 mm vs. 16.69 ± 1.06 mm in *C. alexandrinus*, *p* < 0.001, Fig. 1b), longer wings (118.58 ± 2.90 mm vs. 115.59 ± 3.02 mm in *C. alexandrinus*, *p* < 0.001, Fig. 1c), and larger body mass (48.99 ± 3.26 g vs. 47.56 ± 3.73 g in *C. alexandrinus*, *p* = 0.001, Fig. 1d, Additional file 1: Table S5) than those of *C. alexandrinus*. There was no difference in tarsus length between the two species (*p* = 0.962). Individuals of *C. alexandrinus* from Taiwan Island were heavier than continental populations of *C. alexandrinus* and *C. dealbatus* (both *p <* 0.001). *Q*_*ST*_ between coastal populations of *C. alexandrinus* and *C. dealbatus* was negatively correlated with geographic distance (R^2^ = 0.293, *p <* 0.001, Fig. 1g).

### Genetic diversity and population structure

**Fig. 2:**
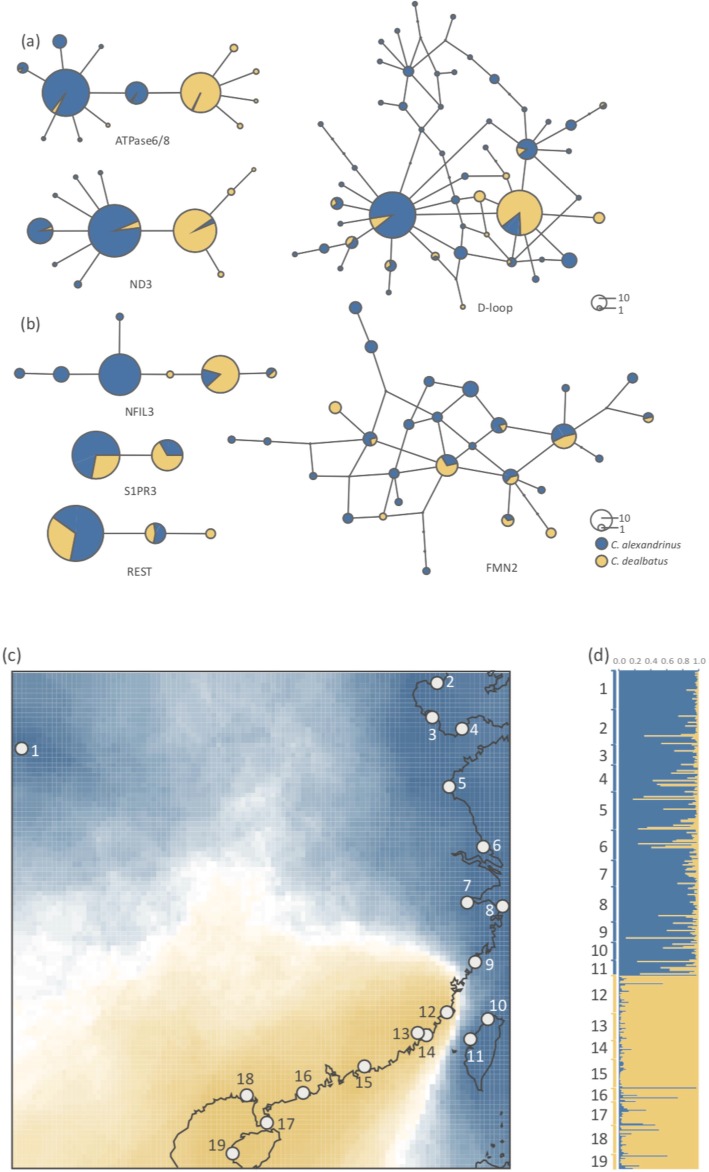
Haplotype networks based on mitochondrial and nuclear DNA sequences, and population genetic structures based on microsatellite loci. *C. alexandrinus* is marked in blue and *C. dealbatus* in yellow. **a** Haplotype networks of three mitochondrial loci. For ATPase6/8 and ND3, over 95% of the individuals are sorted into two major haplogroups. **b** Examples of haplotype networks based on four exonic nuclear loci (NFIL3 and S1PR3 are Z-linked loci) of 55 to 80 individuals. **c** Genetic clustering inferred from microsatellite genotypes using Geneland. Blue and yellow bars show the assignment probability of a location for alternative genetic clusters. Location numbers are consistent with Fig. 1. **d** Genetic clustering inferred from microsatellites using STRUCTURE. Map source: National Geomatics Center of China (http://www.ngcc.cn)

We sequenced 357 individuals at all three mtDNA loci (224 *C. alexandrinus* and 133 *C. dealbatus,* GenBank Accession No. MK830738-MK830815). For each site, the sample size ranged from 11 to 30 individuals. For each individual, we obtained in total 1729 base pairs (bp) of mtDNA sequence, including 846 bp ATPase6/8, 505 bp D-loop and 378 bp ND3. *C. alexandrinus* showed higher intraspecific genetic diversity than *C. dealbatus* (Table 1). Haplotype networks show that at all loci, most individuals were sorted into two major haplogroups, corresponding to the two species of plovers. One non-synonymous substitution separated the two haplogroups at both ATPase6/8 and ND3 loci (Fig. 2a). Moreover, a subset of samples containing 20 individuals of each species were sequenced at 16 loci (range 440–902 bp for each locus; Table 1 and Additional file 1: Table S1, GenBank Accession No. MK830816-MK830957) for a total of 11,209 bp of nuclear DNA sequence. The haplotype networks from autosomal and Z-linked loci did not show strong patterns of lineage sorting like the mtDNA. The most common haplotypes were shared by both species of plover (Fig. 2b and Additional file 1: Figure S1). Moreover, both species showed signs of recent demographic expansion as detected by significant Tajima’s *D* values (Table 1).

**Table 1:** Sampling localities and genetic polymorphism of *C. alexandrinus* and *C. dealbatus*. The number of individuals (*n*) analyzed for mtDNA, autosomal microsatellites and nuclear exonic loci (nuDNA) are given. Site number corresponds to the numbers in Fig. 1. Estimates of *h*, number of haplotypes; *Hd*, haplotype diversity; *π*, nucleotide diversity; *Tajima’s D* value*, Ho*, observed heterozygosity; *He*, expected heterozygosity were calculated for each locality and species

Site		Latitude	Longitude	mtDNA					Microsatellites			nuDNA	
				*n*	*h*	*Hd*	*π*	*Tajima’s D*	*n*	*Ho*	*He*	*n*	*Tajima’s D*
*C. alexandrinus*				224	86	0.929	0.00181	−1.863*	219	0.723	0.802	30–40	0.216
1	Qinghai Lake	36.693	100.758	30	13	0.846	0.00119	−1.785	30	0.703	0.756	13–22	
2	Tangshan	39.169	118.772	23	16	0.945	0.00171	−1.434	30	0.797	0.800	12–20	
3	Cangzhou	38.471	117.682	14	8	0.868	0.00097	−0.871	16	0.774	0.791		
4	Weifang	37.131	119.484	21	14	0.895	0.00158	−1.687	22	0.671	0.787		
5	Lianyungang	34.742	119.243	28	20	0.963	0.00189	−1.166	30	0.697	0.777		
6	Nantong	32.575	121.043	25	12	0.913	0.00185	−0.016	26	0.731	0.796		
7	Zhoushan	29.991	122.151	19	11	0.924	0.00111	−0.141	20	0.746	0.779		
8	Ningbo	30.205	120.918	25	18	0.963	0.00179	−0.958	29	0.751	0.793		
9	Wenzhou	27.937	120.919	14	13	0.989	0.00161	−1.048	16	0.740	0.768		
10	Xinbei	25.862	119.614	14	6	0.736	0.00108	−0.971	30	0.672	0.700	2–8	
11	Zhanghua	24.559	118.297	11	5	0.709	0.00076	−0.893	22	0.692	0.669	4–8	
*C. dealbatus*				133	29	0.667	0.00086	−1.989*	131	0.714	0.758	23–28	0.326
12	Fuzhou	25.190	121.392	29	12	0.874	0.00108	−1.445	15	0.718	0.761	5–8	
13	Xiamen	24.162	120.402	18	6	0.699	0.00075	−1.532	12	0.755	0.748		
14	Jinmen	24.448	118.600	13	7	0.731	0.00110	−1.362	15	0.733	0.703		
15	Shanwei	22.798	115.418	15	3	0.590	0.00039	0.221	23	0.669	0.696		
16	Yangjiang	21.589	111.758	10	5	0.822	0.00158	−0.144	11	0.713	0.718		
17	Zhanjiang	20.238	109.921	18	7	0.569	0.00099	−1.696	20	0.719	0.721		
18	Beihai	21.426	109.196	18	4	0.314	0.00044	−1.849*	23	0.692	0.695	18–12	
19	Dongfang	19.234	109.804	12	2	0.167	0.00019	−1.141	12	0.763	0.679		
All				357	109	0.922	0.00216	−1.741	402	0.719	0.780	55–80	0.271

Overall genetic differentiation was significant and high in mtDNA data (*Φ*_ST_ = 0.506, *p* < 0.001) and low at microsatellite loci (*F*_ST_ = 0.036, *p* < 0.001). For nuclear sequence data, genetic differentiation between species was also significant at autosomal loci (*Φ*_ST_ = 0.100, *p* < 0.001) and particularly high at the Z-linked loci NFIL3 (*Φ*_ST_ = 0.726, *p* < 0.001) and S1PR3 (*Φ*_ST_ = 0.309, *p* < 0.001). In the AMOVA analysis, we observed the largest difference between groups when the data were partitioned by species (i.e. *C. alexandrinus* and *C. dealbatus*), with this grouping explaining 49.7% of the variance in mtDNA and 2.4% in the microsatellites (*p* < 0.001; Table 2). These were significantly higher than the values of genetic variation observed when we partitioned samples as coastal vs. island populations or coastal population vs. Qinghai vs. Taiwan populations. Within *C. alexandrinus*, minor genetic differentiation was found between the inland population (Qinghai Lake) and coastal populations (mtDNA *Φ*_SC_ = 0.042, *p* = 0.022; mst *F*_SC_ = 0.021, *p* < 0.001). *C. alexandrinus* populations on Taiwan Island also shared haplotypes with other coastal populations (mtDNA *Φ*_SC_ = 0.021, *p* = 0.146), but were significantly differentiated in microsatellites (microsatellite *F*_SC_ = 0.016, *p* < 0.001).

**Table 2:** Hierarchical analyses of molecular variance (AMOVA) based on concatenated mtDNA data and 13 microsatellite loci for *C. alexandrinus* and *C. dealbatus*. Samples were partitioned in three groupings: 2 groups (*C. alexandrinus* and *C. dealbatus*); 3 groups (*C. alexandrinus* continental populations and Taiwan island populations, *C. dealbatus*); 4 groups (*C. alexandrinus* inland (Qinghai Lake), *C. alexandrinus* coastal, *C. alexandrinus* Taiwan island, and *C. dealbatus*). *Va*, genetic variation among groups; *V*b, variation among populations within groups; *V*c, variation within populations. Between-group genetic differentiation was highest when populations were partitioned into two groups (*C. alexandrinus* and *C. dealbatus*)

	Grouping	*Va*/%	*Vb*/%	*Vc*/%
mtDNA	*2	*49.712	*8.491	*41.797
	3	45.695	9.269	45.036
	4	41.782	10.296	47.922
microsatellites	*2	*2.416	*1.798	*95.786
	3	2.192	1.749	96.059
	4	1.917	1.776	96.307

All *p < 0.001

For microsatellite loci, 18 out of 22 markers were successfully genotyped. However, four markers (Calex-04, 08, 19, C204) showed a large proportion of missing data, while another four loci (Calex-11, 24, 26, 43) showed an estimated frequency of null alleles over 10%. Moreover one locus (Calex-35) showed H-W disequilibrium (Additional file 1: Table S2). Consequently, the aforementioned nine loci were removed from the dataset, so the final microsatellite dataset used for further analysis consisted of the genotypes of 402 individuals (271 *C. alexandrinus* and 131 *C. dealbatus*) at 13 different loci.

The results of the STRUCTURE analysis clearly showed two genetic clusters representing each species (Fig. 2d) and no obvious gradual transition along the coastline that would be expected from a hybrid zone. The average *Delta K* value when *K* = 2 was much higher than that of other options (Additional file 1: Figure S2). Using georeferenced data in GENELAND, our results corroborated the genetic clustering patterns inferred from STRUCTURE. The posterior probability was 0.70 when *K* = 2, which contrasted with a value of 0.25 when K = 3. We visualized the GENELAND results on a map with the probability distribution of posterior mode of class membership, which further supports the separation between *C. alexandrinus* and *C. dealbatus* along the China coast (Fig. 2c). The divide between these two species was located between Wenzhou and Fuzhou, according to the GENELAND results, but it is unclear if these two species come into contact or form a putative hybrid zone in this region. Again, the individuals from the sites in Taiwan were assigned to the cluster of *C. alexandrinus* (Fig. 2c).

### Inferred demographic history

Isolation with migration analyses suggested that *C. alexandrinus* and *C. dealbatus* diverged 0.56 (0.41–5.19) million years ago. Estimated migration rates in both directions were significant (*p* < 0.001) with slightly higher gene flow from *C. alexandrinus* into *C. dealbatus* (2.69 migrants per generation) than vice versa (ca. two migrants per generation). The estimated effective population size of *C. alexandrinus* (*N*_e_ ≈ 1.59–4.44 million) was about 8–10 times higher than for *C. dealbatus* (N_e_ ≈ 0.15–0.52 million).

The estimation of recent gene flow between the two species, carried out with BayesAss using microsatellites, also suggested bidirectional gene flow. Gene flow from *C. dealbatus to C. alexandrinus* was slightly higher (0.013, *p* = 0.028) than in the other direction (0.010, *p* = 0.028). In *C. alexandrinus* populations, one likely migrant from *C. dealbatus* and two hybrid F1 individuals were identified (probability higher than 0.5). With the same threshold value, two migrants and one hybrid F1 individual were identified in *C. dealbatus* populations.

### The detection of hybrids

Based on the STRUCTURE results and simulations, the optimal threshold for distinguishing hybrids from non-hybrids was q = 0.836. Based on this threshold, 81.9% of the individuals (204 out of 246) collected from sites at the northern Chinese coastline, Qinghai Lake and Taiwan Island were assigned to *C. alexandrinus* (Fig. 2c, d)*.* Individuals with intermediate q-values, which were possibly hybrids, were found at most northern Chinese sampling sites (Fig. 2d). For the southern coastline in China, 145 out of 156 Individuals (92.9%) belonged to *C. dealbatus* with a probability larger than 0.836 (Fig. 2c, d). Only one individual of each species was assigned to the a migrant from the other species with high probability (Fig. 2d).

### Ecological niche profiles

**Fig. 3:**
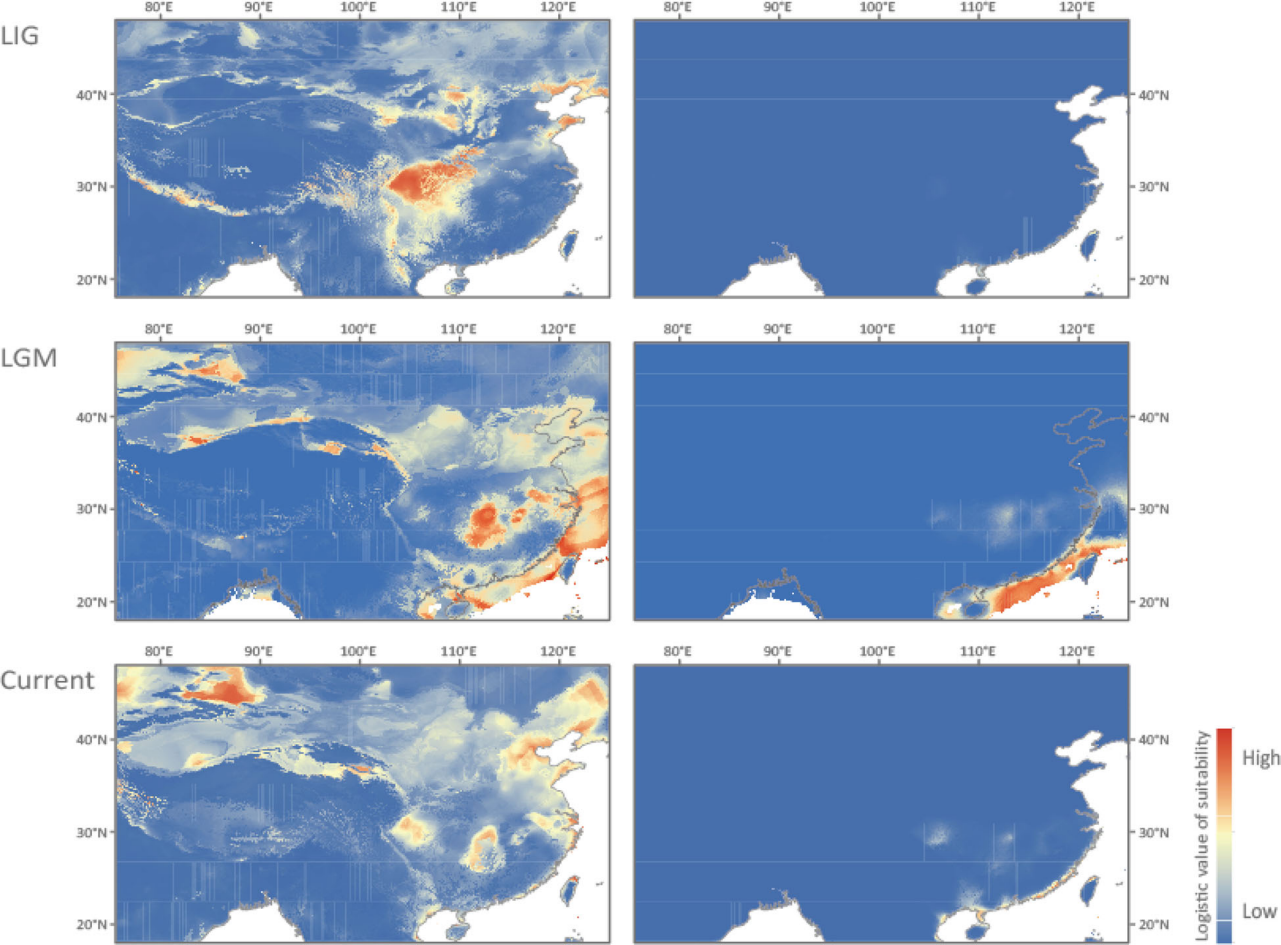
Predicted environmental suitability for *C. alexandrinus* (left) and *C. dealbatus* (right), via ecological niche modeling (ENM). ENM results are shown for the Last Interglacial (LIG, 120-100Ka), Last Glacial Maximum (LGM, 21Ka, MIROC model) and current time periods, respectively. Maps were drawn based on the projected distributions using ArcGIS 9.3 (ESRI, Redland, CA. URL http://www.esri.com/)

Our ecological niche models effectively captured the current distribution of both *C. alexandrinus* and *C. dealbatus* (Fig. 3) with a high discrimination capacity (AUC values > 0.88 for training and test data). Jackknife tests on variable importance for *C. alexandrinus* revealed that isothermality, precipitation seasonality and mean temperature of the warmest quarter were the three highest ranked variables when used in isolation. For *C. dealbatus*, mean diurnal range and annual precipitation were the most important variables. The simulation of the three periods, i.e. Last Interglacial (LIG, 120-100Ka), Last Glacial Maximum (LGM, 21Ka, MIROC model) and current times, respectively, showed range shifts in both species. In particular, these results suggest that the ranges of both species shrank during the LIG in the Chinese coastal area accompanied by an increase in climatic suitability in the inland region for *C. alexandrinus*. In contrast, suitable habitats expanded for both species during the LGM in the coastal area, the East China Sea and the northern part of the South China Sea (sea between Hainan and Taiwan), probably due to a fall in sea level.

The ordination approach, using PCA-env, suggested that the overlap of the current climatic niches of the two species is relatively low (Additional file 1: Figure S3a, b). The two species can be separated based on the first two PCs with an accumulative 81.5% of the total variance explained (Additional file 1: Figure S3c). The niche equivalency test rejected the null hypothesis that the species pair is distributed in identical environmental space (*p* = 0.019; Additional file 1: Figure S3d).

### Stable-isotope profiles

**Fig. 4:**
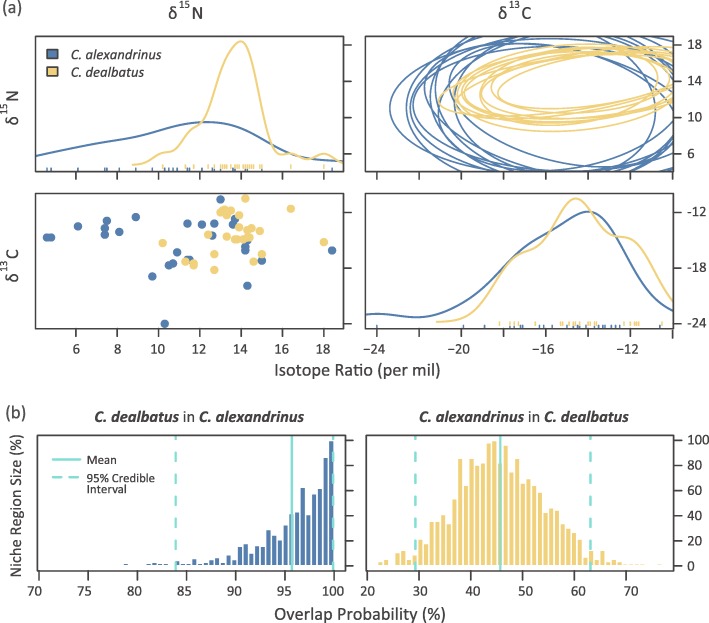
**a** Raw data (bottom-left), density distribution (top-left and bottom-right), and stable isotopes related to dietary niche regions (top right) of δ^13^C and δ^15^N profiles of *C. alexandrinus* and *C. dealbatus* feathers generated by “nicheROVER” (Swanson et al. 2015). The niche region of *C. alexandrinus* (blue) contained that of *C. dealbatus* (yellow) and was the broader of the two regions. **b** Distribution of the posterior probability that an individual from one species is found within the niche region of the other species. Vertical lines for mean and 95% credible intervals are included in the histogram of each overlap metric

Our stable-isotope analysis showed that *C. dealbatus* exhibited significantly higher δ^15^N values than *C. alexandrinus* (*p* < 0.001, Fig. 4a, Additional file 1: Figure S4). In contrast, we did not find significant differences in δ^13^C between the two species (*p* = 0.161). Further isotope space overlap analysis showed that *C. alexandrinus* individuals had a high probability of being found within the isotopic niche space of *C. dealbatus* (95.3%), while *C. dealbatus* individuals showed a relatively low probability of being found in the isotopic niche space of *C. alexandrinus* (46.5%, Fig. 4b). Moreover, we found that *C. alexandrinus* showed higher variability in δ^15^N profile across breeding populations than *C. dealbatus.*

## Discussion

Our genetic data show that *C. alexandrinus* and *C. dealbatus* have diverged to a level of advanced allele sorting between the two species, particularly in mtDNA and Z-linked genes with lower effective population sizes (Fig. 2a, b). For autosomal microsatellite data, we also found that genetic differentiation is low at the intraspecific level but substantially high between the species (Fig. 2c, d, Table 2). Our results suggest the two plover species diverged approximately 0.6 mya, and both have large effective population sizes. Though we found no or just a narrow hybrid zone exists in the contact area on the Chinese coast, a considerable level of symmetric gene flow occurred between the two plovers (Table 3). Further, we found significant differences in morphometric traits and ecological characters between the two plovers along the coast (Fig. 1e-g, 3 and 4).

**Table 3:** Posterior mode, mean and range of 95% highest probability distribution (HPD) of six demographic parameters inferred with IMa2p between *C. alexandrinus* and *C. dealbatus.* Divergence times (*T*) are given in million years ago (Ma). The effective population size (*Ne)* of *C. alexandrinus* was about eight times that of *C. dealbatus.* Migration rate (*2NM*) into each species was about the same. K represents Kentish Plover *C. alexandrinus*; W represents White-faced Plover *C. dealbatus*; A is the most recent common ancestor of two species

Parameter	*T*/Ma	*Ne*_*w*_/10^6^	*Ne*_*k*_/10^6^	*Ne*_*A*_/10^6^	*2NM*_*K- > W*_	*2NM*_*W- > K*_
Mode	0.557	0.259	2.036	0.495	2.696*	2.005*
Mean	2.160	0.297	2.450	0.589	2.859	4.306
2.5% HPD	0.413	0.154	1.590	0.134	1.581	0.528
97.5% HPD	5.187	0.515	4.442	1.098	4.503	11.500

* *p* < 0.001

### Phylogeographic patterns

**Fig. 5:**
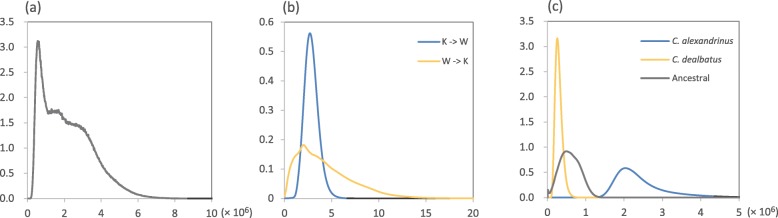
Posterior densities of population demographic parameters estimated using the isolation-with-migration model (IM) implemented in IMa2p. These analyses used the combined dataset of three mitochondrial (1729 bp) and 15 nuclear exonic loci (11,209 bp). K represents the Kentish Plover *C. alexandrinus*; W represents the white-faced Plover *C. dealbatus*. **a** Population divergence times (*T*) of *C. alexandrinus* and *C. dealbatus*. **b** Population migration rates (*2NM*) represent the average number of individuals in each group that had previously migrated from the other group. Gene flow in both directions was significant. **c** Effective population sizes (*Ne*) of the two species and their most recent common ancestor

Two genetic lineages were found among breeding *Charadrius* plovers along the Chinese coast, the northern lineage corresponding to *C. alexandrinus* and the southern one to *C. dealbatus* (Fig. 1a, Table 2). The sharp genetic break between the two lineages lies between Wenzhou and Fuzhou (latitude 26–27 °N), north of the Taiwan Strait (Fig. 2c, d). Furthermore, samples from Taiwan Island belong to *C. alexandrinus* but the population in Jinmen Island (site 14 in Fig. 1a), off Fujian coast is affiliated with *C. dealbatus* (Fig. 2c, d). Our IMa analysis estimated that the divergence time between the two plovers was back to approximately 0.6 mya (Table 3, Fig. 5), which falls into Marine Isotope Stage 16 of the mid-late Pleistocene climatic fluctuation periods [50]. For ocean marginal and coastline taxa, this implies that fluctuations in sea level can lead to alternation between population isolation and contact throughout their evolutionary histories. Because *Charadrius* plovers originated in the Northern hemisphere and then radiated to the Southern hemisphere [51], it is thereby likely that *C. dealbatus* evolved from a recent common ancestor with *C. alexandrinus* and could have been diverging along the east China coast since the mid-late Pleistocene. During this period, vicariance occurred because the coastline was separated by a land-bridge raised due to the shallow sills between the East and the South China Sea [52]. As shown in our niche modeling analysis, both species had decreased distribution ranges during the LIG followed by enlarged suitable habitats and overlapping ranges between the East and South China Sea during the Last Glacial Maximum (Fig. 3). While our niche modeling demonstrates a cycle of range dynamics caused by sea-level changes, one should bear in mind that the last million years witnessed multiple glaciation cycles [53, 54]. It is possible that the divergence between the two plovers proceeded through cycles of allopatric stages interspersed by secondary contacts due to sea-level fluctuation.

To the best of our knowledge, our study shows the first documented phylogeographic break in a bird species along the coastline of China. Phylogeographic patterns have been relatively well characterized in shorebirds breeding at high latitudes [32–34]. Panmixia or weak genetic differentiation was usually suggested, most likely driven by extensive gene flow. However, it seems that islands can act as a barrier to natal dispersal for the continental Kentish Plovers [32]. In contrast, population structures in temperate and subtropical shorebird populations are poorly documented, probably due to a low level of species diversity. Interestingly, concordant phylogeographic patterns have been described in several coastal marine taxa, such as plants [55], fishes [56, 57], shellfishes [58], and crustaceans [59]. Though the exact splitting times are not congruent, the observed split line is at approximately 25°N latitude between the East and the South China Sea (reviewed in [52]). This pattern resulted in the hypothesis that historical factors, i.e. sea level fluctuation during the Pleistocene, caused a convergent phylogeographic pattern in multiple coastal marine fauna in the marginal northwestern Pacific Ocean [52, 56]. Thus, our study contributes a vertebrate case to the accumulating literature about this hotspot of species divergence. Apart from the major role of physical barriers, comparative phylogeographic studies also revealed that other abiotic factors, like ocean currents and hydrothermal conditions, as well as the ecological characters of species, i.e. dispersal ability, habitat preference, life-history, and population demography, can also play a role in contributing to the divergence of coastline fauna [52, 57].

### Phenotypic and ecological differentiation along a latitudinal gradient

The two plovers, *C. alexandrinus* and *C. dealbatus*, are distributed along the Chinese coastline across latitudinal and associated environmental gradients from temperate to tropical zones. Within each species, we found a general trend that northern populations have larger morphometric values when compared with their southern counterparts (Fig. 1). This pattern may be related to Bergmann’s rule which states that the body size of homeothermic animals is larger in colder climates than in warmer ones [60]. Populations of both plover species may benefit from optimal control due to their distribution across such a north-south gradient [61]. A mutually non-exclusive explanation is that the difference in morphological traits, i.e. body mass and bill length, link to potential differences in resource exploitation and life history. Our data show that *C. dealbatus* has a larger average body mass than *C. alexandrinus.* Body mass is a comprehensive trait reflecting nutrition assimilation, energy reservation and expenditure [62]. The difference in body mass might be related to the difference in migratory behavior where a lighter body mass in *C. alexandrinus* is favored by decreased transport cost of fuel storage during migration [63, 64]. In contrast, larger body mass in *C. dealbatus*, a short-distance migrant or resident, may be beneficial for multiple reproductions within a single breeding season while the lighter *C. alexandrinus* usually only produces a single clutch (Lin et al. in prep.). The difference in bill length may be driven by the difference in the use of food resources and also foraging strategies [65] but recently, the function of the bill as a temperature regulator has also started to attract scientific attention [66]. An increasing number of studies have demonstrated that birds at higher latitude and in cooler environments have shorter bills (European sparrows, [67], Australian shorebirds, [68]), consistent with Allen’s Rule [69]. Nevertheless, we found that populations of *C. alexandrinus* in Taiwan had similarly large body mass and bill lengths when compared to *C. dealbatus* populations (Fig. 1b-d, g), which are significantly larger than mainland conspecific populations. This is probably caused by phenotypic plasticity of the Taiwan population towards sub-tropical ecomophology and a resident life history, similar to the populations of *C. dealbatus* in the South China Sea coast.

The niche-equivalency test in environmental (E)-space rejects the possibility that the two species are distributed in strictly equivalent environmental space (Additional file 1: Figure S3). Corresponding to this, in geographical (G)-space, *C. dealbatus* is restricted to breeding sites close to the coast, particularly where there is a warmer tropical climate. In contrast, *C. alexandrinus* has a wider climatic niche, as represented by a broader climatic zone (Fig. 3). This species can breed not only on temperate coasts [70] but also on inland saline lake shores [71], such as Qinghai Lake. In addition, our isotope analysis revealed that *C. alexandrinus* covered a wider range of isotope ratios than *C. dealbatus*, but *C. alexandrinus* exploited a lower trophic range (δ^15^N). This indicates that *C. dealbatus* probably feeds on a higher energy diet than its sister species [72]. However, it is unclear whether such differences result from divergence in diet preference or food resource availability [73]. Taken together, these results suggest that the ecological niches of the two plovers are significantly different in several aspects, and support a role for ecology in constraining the range limits, and perhaps promote reproductive barriers between the two shorebird species.

### Molecular signatures of early species divergences in two plover species

After the two nascent plover species split, how was their divergence maintained in the presence of gene flow? Speciation theory predicts that divergence is initiated either by genetic drift or divergent selection [22, 74, 75]. However, genetic drift is unlikely to have been the only force to initiate divergence in the plovers. Given the large effective population sizes (*Ne*) and recent divergence (Table 3), it is difficult that genetic drift could have led to fixed differences between populations [76, 77]. Gene flow can directly counteract the diverging effects of drift [78, 79] but sufficient selection could help overcome the homogenizing effects of gene flow [1–5]. We found some hints of selection by comparing the level interspecific genetic differentiation among genetic markers. For sequence data, the ratio of *Φ*_ST_ was five times larger at the concatenated mtDNA dataset, and 3~7 times larger at the Z-linked genes than at the autosomal loci, respectively. Under migration-drift equilibrium, one would expect that divergence should be four and 1.33 times larger than the extent of genetic diversity at autosomal markers, respectively [80, 81], and thus the observed elevated extent of genetic divergence for mtDNA, and especially for Z-linked markers cannot be explained by genetic drift alone. For maternally inherited mtDNA, both female philopatry and selective sweeps in the mitochondrial genome could cause the deviation from the neutrality model [24]. However, a high level of female philopatry is less likely due to evidence of female-biased dispersal suggested by field observation and genetic inference in *C. alexandrinus* [24]. Thus selection on mitochondrial DNA could explain the observed pattern, and this might be further supported by the significantly negative values of Tajima’s *D* (Table 1) throughout all study populations.

In addition, elevated levels of genetic divergence are often found at Z-linked markers compared with autosomal markers, especially at the nascent stage of speciation, termed as the faster-Z effect [82]. Indeed this pattern has been shown in several empirical studies in birds [reviewed in 125]. Beside neutral explanations, such as the lower *N*e (3/4 that of the autosome) and higher mutation rate than autosomes, the Z chromosome has been demonstrated to contribute to reproductive isolation by playing a large role in promoting both prezygotic and postzygotic isolation, because some regions in the Z chromosome can be responsible for encoding sex-related or mating preference traits [83]. Hence divergent selection or ecologically-mediated sexual selection can reduce genetic introgression on the Z chromosome and play a disproportional role in the divergence in birds, but the current data do not have enough power to resolve this link in the plover case.

The loci analyzed here represent just a small proportion of the genome and further investigations using whole genome data hold great potential for providing insights on of the genetic basis of divergence in the two plovers. Particularly, it would be important to identify specific genomic regions (“genomic islands of speciation”) characterized by a high level of divergence and reduced level of gene flow [2–5]. Data from several organisms suggest that incipient speciation can be maintained with divergence at a small number of genomic regions [11, 12, 84–87]. Because several morphological and ecological differences between the two plovers have been characterized in the present study, it is worth to understand whether genomic islands of high differentiation included relevant candidate genes that are involved in reproductive isolation.

The geographical boundary between the two plovers was ambiguously defined in previous studies [41, 42]. Our results show that the discontinuity in genetic structuring (Fig. 2c) and morphometric values (Fig. 1b-d) between the two species is situated at the coastline between Wenzhou and Fuzhou, indicating a contact zone (Fig. 2c). For incipient species, individuals may show a clinal pattern of allele frequencies and morphology at their contact area and form a hybrid zone [53, 88, 89]. Our STRUCTURE results revealed no obvious signs of a hybrid zone (Fig. 2d), and rather indicated sporadic migrants in the respective range of the other species. NewHybrid 1.1 [90] was also used to identify potential hybrid individuals and the result was highly concordant with STRUCTURE. However, NewHybrids failed to identify simulated hybrid individuals and recognized them as migrants (Additional file 1: Figure S5), probably due to incomplete divergence between two species. With the possibility that sampling was not fine-grained enough, the potential hybrid zone should be narrower than the 200 km distance. Another possibility for an apparent lack of a hybrid zone is assortative mating between the two plovers at the contact area [91]. The latter explanation is very possible because populations of the two species close to this region were much more differentiated in morphological traits than the ones farther apart (Fig. 1b-d) and showed ecological segregation (see discussion above). In this case, reproductive [92, 93] and ecological [94, 95] character displacement are called as mechanism to contribute reproductive isolation between the two species. Even with the aforementioned approach to disentangle the heterogeneous landscape of genomes, genomic data alone might not reveal the form of reproductive isolation between the two plovers [23]. It is essential to carry out detailed experiments to test assortative mating in the contact zone between *C. alexandrinus* and *C. dealbatus*.

### Taxonomic implications

Kennerley et al. 2008 [41] recommended that the tropical breeding population, previously defined as the subspecies *dealbatus*, warranted species status, based on differentiation in morphology, behavior and distribution from *C. alexandrinus* [41]. Uncertainty about the taxonomic status arose because the first genetic evaluation found no evidence of genetic differentiation between the two plovers [42]. At odds with this, the present dataset is comprised of a larger suite of genetic markers and well- characterized ecological traits (Fig. 1), obtained by systematic sampling along the Chinese coast, while Rheindt et al. 2011 [42] analyzed samples collected outside the breeding season. Using the same mtDNA markers we found interspecies divergence not detected in Rheindt et al. [42]. Such underestimated genetic differentiation may be due to the erroneous species assignment of non-breeding birds, because the distinguishing features in non-breeding plumage are relatively subtle [41–43] and non-breeding *C. alexandrinus* and *C. dealbatus* mix frequently on the coast of the South China sea [41]. We further found a very small number of hybrids, and a lack of broad hybrid zone, implying some mechanisms to retain species boundary. Furthermore, we provide new data to support divergent morphometric characters and distinct ecological niches, which are likely key factors for maintaining species limits. Although we did not apply explicit Bayesian species delimitation analyses (e.g. [96]), our results offer compelling evidence that *C. dealbatus* is a distinct lineage from *C. alexandrinus*, and deserves a full species status under the General Lineage Concept of Species (GLC hereafter) [97, 98], nowadays widely adopted by taxonomists. The key essence of GLC is that species are defined as independently evolving metapopulation lineages, and can be assessed from an integrative taxonomic approach [99]. Beyond resolving the taxonomic puzzles between the two plovers, this study forms a basis to allow *C. dealbatus* to be considered as a target for conservation. Because it has a restricted range and is one of few breeding shorebirds in subtropical China coastline, where habitat loss may cause a risk of population decline [100].

## Conclusions

Resolving the balance between diverging selection and gene flow is of fundamental importance to understand speciation processes. Here, we show that *C. alexandrinus* and *C. dealbatus* represent a case of incipient species in which divergent selection associated with ecological differences likely works as an efficient mechanism for the maintenance of divergence in the face of gene flow. While the full species status of *C. dealbatus* may be justified under the General Lineage Concept of Species, it remains untested whether a strong level of reproductive isolation has been established between *C. alexandrinus* and *C. dealbatus*. Furthermore, we have shown low genetic divergence between the two plovers, so it would be of particular importance to explore the patterns of divergence at the genome level and determine whether specific regions are related to reproductive isolation and adaptation. In addition, the IM analysis presented here estimated average historical gene flow, but it would be interesting to evaluate more realistic (and complex) demographic models in order to better estimate changes in *Ne* and the span of isolation and secondary contact. Genome-wide population genetic approaches promise the power to resolve the evolutionary history of the two *Charadrius* plovers but also open an avenue to characterize the genetic architecture associated with phenotypic trait divergence and local adaptation [84, 101].

## Methods

### Sampling

We mainly collected samples along the eastern coastal area of China (Fig. 1a), and also obtained samples from the two biggest continental islands, Taiwan and Hainan, as well as two small islands that are close to the Chinese coastal line (Zhoushan and Jinmen). Sampling sites were separated by approximately 200–250 km along a 2300 km transect, spanning almost the entire Chinese coastline. Furthermore, one high altitude population (breeding at 3350 m above sea level) near Qinghai Lake was sampled as an inland outgroup. Breeding individuals were captured using the funnel-trap method described in [102] between March and July in 2014–2015. For each nest in each locality, blood samples of the breeding pair in the most cases or a single chick were collected. Tissue samples were also obtained from dead individuals found in the field. Species identification performed during sampling was based on the summary of plumage characters as well as morphometric and ecological differences between *C. alexandrinus* and C. *dealbatus*, as described by [41]. Birds were released after morphometric measurement and blood collection. Overall, we collected samples from 454 individuals from 19 breeding sites.

### Morphometrics

For each adult individual, four basic morphometric measurements were taken: body mass, wing length, tarsus length and bill length. Body mass was measured with an electronic scale (± 0.1 g). Wing length (flattened) was measured with a wing ruler (± 1 mm). Bill length to skull and tarsus lengths were measured using vernier callipers (± 0.1 mm). Measurements were taken by Q.H., P.J.Q. and C.Y.C. following the standard described in [103]. To avoid potential biases, some individuals were measured twice or three times by different authors between 20 and 30 trials to make sure there was no significant measurement difference (*p* < 0.05,) at the beginning and end of the fieldwork in the year 2014–2015.

We carried out principal component analysis (PCA) for the four traits to visualize the variation of morphometric data. Analyses of covariance (ANCOVAs) were conducted based on the PCA results to test statistical significance. We carried out t-tests for each measurement to assess the difference between species. Pairwise morphological difference *Q*_*ST*_ based on PC1 scores from PCA were calculated following the method used by JF Storz [104] and plotted against linear distances between coastal populations. All aforementioned analyses were performed in PAST 3.12 [105].

### Molecular genetic methods

We extracted genomic DNA using Tiangen Blood & Tissue Genome DNA Kits, following the manufacturer’s protocols (Tiangen, Beijing, China). DNA quality was measured with a NanoDrop 2000 (Thermo Scientific, USA). Three mtDNA loci, namely partial ATPase subunit 6/8, partial D-Loop of the mitochondrial control region (CR) and NADH dehydrogenase subunit 3 fragment (ND3), were amplified from all samples using primers from [42]. PCR reactions for mtDNA amplification were carried out in 20 μl volumes containing 1X PCR buffer (Takara Shuzo, Japan), 10–50 ng DNA template, 0.5 U Taq DNA Polymerase (Takara Shuzo, Japan), 1.0 μM of each primer, 2.0 mM of each dNTP and 1.0 μM MgCl_2_. We also sequenced 14 autosomal and two Z-linked exonic loci [106] for a subset of 40 individuals (Table 1) representing both species and several populations within species. PCR for nuclear loci was performed using a higher concentration of dNTP (4.0 mM) and MgCl_2_ (2.5 μM). The PCR cycling profiles used are listed in Additional file 1: Table S3. Each PCR product was checked on a 1% agarose gel prior to being sequenced on an ABI3730XL (Applied Biosystems, USA) by MajorBio, Shanghai, China.

Additionally, all samples were genotyped for 22 autosomal microsatellite markers (mst), mostly those genotyped by Küpper et al. [107], but also the markers C204 [108] and Hru2 [109], respectively. Microsatellite loci were amplified using three multiplex PCRs with respective cycling profiles (Additional file 1: Table S3). Each PCR was in a total volume of 15 μl containing 1X HotStart buffer (Tiangen), 10 ng DNA template, 1 unit Multi HotStart DNA Polymerase (Tiangen), 0.4 μM of each fluorescently labeled primer, 2.0 mM of each dNTP and 2.0 μM MgCl_2_. Multiplex PCR products and associated genotypes were isolated on an ABI3730XL (Applied Biosystems, USA) by Invitrogen, Shanghai, China and their length was determined using GeneMapper software v.3.7 (Applied Biosystems) against an internal size standard (GeneScan-500LIZ; Applied Biosystems).

### Population genetic analyses

For DNA sequence data, we aligned each mitochondrial or nuclear locus using the CLUSTAL W algorithm in MEGA v.6.06 [110], and the alignment was checked by eye and manually edited if needed. For nuclear DNA sequence data, we first used PHASE 2.1.1 [111] to reconstruct haplotypes of nuclear sequences with heterozygous sites. Each run was set to 10,000 iterations, 100 burn-in and 10 thinning intervals. For both mtDNA and nuclear loci, basic genetic polymorphism statistics, such as haplotype number (*h*), haplotype diversity (*Hd*), number of segregating sites (*S*), nucleotide diversity (*π*) and Tajima’s *D* [112], of each locus and each population were calculated in DnaSP 5.10.1 [113]. Haplotype networks of each locus were constructed using a median-joining algorithm [114] in PopART 1.7.2 [115].

We used FreeNA [116] to check for the frequency of null alleles at each microsatellite locus. Further tests for Hardy-Weinberg equilibrium and pairwise linkage disequilibrium (LD) were carried out with Arlequin 3.1.1 [117]. Hardy-Weinberg equilibrium tests were run with 10,000 permutations. LD tests were run for 100,000 steps of the Markov chain. To obtain genetic diversity estimates, we calculated observed heterozygosity (*H*o) and expected heterozygosity (*H*_E_) in GenAlEx 6.5.1 [118].

We estimated population structure between species and among sampling sites within species with several approaches. First, for the concatenated mtDNA sequences and microsatellites, we performed analyses of molecular variance (AMOVAs) in Arlequin to assess the proportion of genetic variance explained by the different partition settings: the two species; *C. alexandrinus* continental populations and Taiwan island populations; *C. dealbatus*, and coastal populations (including Hainan Island), Qinghai and Taiwan. Second, we also calculated pairwise *Φ*_ST_ and *F*_ST_ between breeding sites for mtDNA and mst, respectively, and we derived significance levels using 10,000 permutations in Arlequin.

For microsatellite genotypes only, we carried out assignment analyses with two model-based Bayesian approaches. First, we performed a Bayesian clustering analysis using the admixture model with correlated allele frequencies in STRUCTURE 2.3.4 [119, 120]. Ten independent analyses were run from *K* = 1 to *K* = 8 for 500,000 Markov chain Monte Carlo (MCMC) generations with 100,000 burn-in. Replicate runs were combined using STRUCTURE Harvester 0.6.94 [121] and CLUMPP 1.1.2 [122]. The most likely number of genetic clusters was also determined using Structure Harvester using the criteria described in Evanno et al. [123]. Second, we used a Bayesian clustering algorithm, that takes the geographical coordinates of each sampling site into account, using the R package, GENELAND 4.0 [124]. One million MCMC iterations were performed using a thinning setting of 100, from 1 to 10 populations, and a maximum Poisson process rate of 100. The uncertainty of special coordinates was set to zero and the maximum number of nuclei in the Poisson-Voronoi tessellation was 300. An independent Dirichlet distribution model for allele frequencies was also used. With the same package, the most likely number of clusters was determined based on their posterior density. To confirm the consistency of the results, we repeated the MCMC simulation 10 times.

Furthermore, to determine the probability that an individual was a hybrid or a migrant, we estimated the posterior probability of each individual based on microsatellite genotypes. First, we used HYBRIDLAB 1.0 [125] to simulate 100 individuals of each species, F1, F2 and back-crosses in each direction. Microsatellite genotypes of 260 individuals with a q-value higher than 95% in STRUCTURE analysis were used as the genotype pool where the genotypes of simulated parents were drawn from. One hundred simulated parents from each species and 100 F1 were used to calculate the threshold for individual hybrid assignment in STRUCTURE following the method used by J Vähä and C Primmer [126].

### Historical demographic analyses

To infer the demographic histories of the two plover species, we applied Isolation with Migration model (IM) [82] analyses using the combined sequence data set of 16 nuclear loci and the concatenated mtDNA sequences based on 20 individuals of each species. IM analysis allows the inference of genealogies under different demographic scenarios and the estimation of population genetic parameters, such as divergence times, effective population sizes and migration rates between species since their divergence from the common ancestor. The homologous Killdeer *Charadrius vociferus* sequences from GenBank were used as an outgroup. The generation time was set to 2.5 years. The substitution rate of each locus was calculated using the method in Li et al. [127]. The ratio of net genetic distance of each locus across ingroup–outgroup was calculated, compared with net distance of mitochondrial cytochrome b (*cytb*) and then multiplied by the substitution rate for *cytb* (0.0105 ± 0.0005 substitution/site/mya) [128]. We used the Phi test [129] for recombination within nuclear loci and no recombination was detected. We implemented models in IMa2p [130], the parallel version of IMa2 [131]. For each analysis, we ran 48 MCMC chains for 2,500,000 steps of burn-in, after which 500,000 genealogies were saved, each recorded after 100 steps. Because IM analysis only provides estimates of average gene flow since the divergence of the two species, we used BayesAss 3.0.4 to perform a Bayesian analysis based on [132] based on microsatellite genotypes to characterize the level of gene flow within recent generations. We performed 10,000,000 iterations and 1,000,000 steps of burn-in.

### Ecological niche analyses

To infer potential past range shifts induced by climatic changes, we carried out ecological niche modeling (ENM) using Maxent 3.3.3 k [133]. The geographically independent occurrence records of the two species of plovers (93 for *C. alexandrinus* and 24 for *C. dealbatus*) were obtained from our sampling expeditions during 2002–2016, China Bird Report (http://www.birdreport.cn), and the Global Biodiversity Information Facility (GBIF, http://www.gbif.org/). Furthermore, bioclimatic variables were obtained from the WorldClim database v.1.4 [134]. For highly correlated temperature/precipitation variable pairs (|r| ≥ 0.8) [135], we retained the variable with larger contribution to model development and putative greater biological importance (Additional file 1: Table S6). As a result, climatic conditions were measured as a function of eight bioclimatic variables (i.e., the mean diurnal range, isothermality, minimum temperature of the coldest month, mean temperature of the warmest quarter, annual precipitation, precipitation of the wettest month, precipitation of the driest month and precipitation seasonality, see Additional file 1: Appendix S1).

To explore niche similarity between the two species, we performed an ordination null test of PCA-env in environmental (E)-space [136, 137]. We used the methodology previously described by Broennimann and co-workers [136]. This method calculates the occurrence density and environmental factor density along environmental (principal component) axes for each cell using a kernel smoothing method and then uses the density of both occurrences and environmental variables to measure niche overlap along these axes. An unbiased estimate of Schoener’s D metric was calculated for our data, using smoothed densities from a kernel density function to measure niche overlap between the two species to ensure independence from the resolution of the grid. Statistical confidence in niche overlap values was then tested through a one-sided niche-similarity test. All statistical analyses were performed in R 3.0.2 [138]. Details of ENM construction and niche-similarity tests are available in Additional file 1: Appendix S1.

### Stable-isotope analysis

Because the stable isotopic compositions of consumed tissues can be used to estimate the relative contribution of assimilated dietary sources [139], stable-carbon (C) and nitrogen (N) isotope analysis is widely used as a tool to study avian diet and migratory patterns [72, 140]. Carbon isotope ratios differ between C3, C4 and CAM plants due to differences in the photosynthetic pathways, and these differences are incorporated into an animal when the plants are consumed and so can be used to infer information about dietary niches [72] and geographic origins [141]. N isotopes are useful for identifying species/individuals which occupy different trophic positions (high δ^15^N implies higher trophic level [142]). In order to compare dietary differences based on differences in δ^15^N and δ^13^C between the two species, we collected the outer pair of rectrices from seven adults per site at eight sites: Qinghai Lake, Tangshan, Lianyungang and Rudong for *C. alexandrinus*; Fuzhou, Shanwei, Zhanjiang, Dongfang for *C. dealbatus* within our sample set. Since both species perform a complete post-breeding molt within their breeding grounds ([143], personal observation), isotope ratios represent trophic level and habitat preferences during the breeding period. We estimated niche width and overlap per species using an isotopic Bayesian approach based on δ^13^C and δ^15^N profile. Detailed information on lab procedures and statistical analyses can be found in Additional file 1: Appendix S2.

## Additional file

Additional file 1: **Table S1.** Estimates of *h*, number of haplotypes; *s*, number of segregating sites; *π*, nucleotide diversity; *Tajima’s D* value, substitution rate of each locus estimated based on the substitution rate for *cytb* and GenBank accession number for each mtDNA and nuclear locus of *C. alexandrinus* and *C. dealbatus* were provided. The substitution rate of each locus was calculated using the method in Li et al. (2010): the ratio of net genetic distance of each locus across ingroup–outgroup was calculated, compared with net distance of mitochondrial cytochrome b (*cytb*) and then multiplied by the substitution rate for *cytb* (0.0105 ± 0.0005 substitution/site/mya, Weir & Schluter 2008). **Table S2.** Estimates of pairwise *F*_ST_ for each microsatellite locus between *C. alexandrinus* and *C. dealbatus*, LD test result (same for both species), *Na*, number of alleles, *Ho*, observed heterozygosity and *He*, expected heterozygosity in each species. **Table S3.** PCR amplification protocols for three mtDNA, 16 nuclear exons and 22 microsatellite loci genotyped for *C. alexandrinus* and *C. dealbatus.* **Table S4.** Genetic differentiation between each pair of sampling localities of *C. alexandrinus* and *C. dealbatus*. Estimates were based on 1729 bp mtDNA (lower diagonal, *Φ*_ST_) and 13 microsatellite loci (upper diagonal, *F*_ST_). Values highlighted in red represent significant value after Bonferroni correction. **Table S5** The morphological measurements of *C. alexandrinus* and *C. dealbatus* in different breeding sites along the Chinese coast, and Taiwan and Hainan Islands. Site reference number corresponds to numbers in Fig. 1 and Table 1. The number of sample size for each site (n), the mean value of measurements including bill and wing length, body mass and their respective standard deviations (*SD*) are given. Only DNA samples from a breeding site in Cangzhou were collected but not the measurement, the corresponding data is missing for this population. Mean and SD of bill length, wing length and body mass for each population. Measurement data of the site 3-Cangzhou was not collected. **Table S6.** Pearson’s correlation coefficients between pairwise of bioclimatic variables with the ranges of *C. alexandrinus* and *C. dealbatus*. **Figure S1.** Haplotype networks based on 80 individuals of 12 nuclear loci not shown in Fig. 2. *C. alexandrinus* (blue) and *C. dealbatus* (yellow). **Figure S2.** The Bayesian clustering analysis with STRUCTURE clearly suggested two genetic clusters corresponding to *C. alexandrinus* and *C. dealbatus*. Shown is the maximum value of the Delta K (**Δ** K) in posterior likelihood Ln P (X/K) over 10 runs per K of STRUCTURE. **Figure S3.** Niche of *C. alexandrinus* and *C. dealbatus* in climatic space from a principal component analysis (PCA-env). a) and b) show the niche characteristics of *C. alexandrinus* and *C. dealbatus*, respectively, along the first two axes of the PCA. Grey shading shows the density of the occurrences of the species by cell. The solid and dashed contour lines illustrate, respectively, 100 and 50% of the available (background) environment. c) The contribution of the variables on the first two axes of the PCA and the percentage of inertia explained by the two axes. d) Observed niche overlap D between the two ranges (bars with a diamond) and simulated niche overlaps (grey bars) on which tests of niche equivalency were calculated with 100 iterations. **Figure S4.** Differentiation in the stable isotope ratios among breeding sites (top-left: δ^13^C and bottom-left: δ^15^N) and between the two plovers (top-right: δ^13^C and bottom-right: δ^15^N). In all representation, *C. alexandrinus* marked in blue and *C. dealbatus* in yellow. **Figure S5.** (a) Newhybrid simulations failed to reliably detect simulated hybrid individuals. Real data of individuals with posterior density higher than 95% in STRUCTURE, and data of 5 simulated F1 and F2 individuals, and 10 back-cross individuals on each direction was used to imitate a situation when hybrids were the minority in the population. (b) Newhybrid results from real data are highly consistent with the STRUCTURE results. KP represents *C. alexandrinus*, WFP represents *C. dealbatus*, bx represents back-crosses. **Appendix S1.** Reconstruction of potential range shifts induced by climatic changes and inference of environmental niche overlap between the two-plover species, *Charadrius alexandrinus* and *C. dealbatus*. **Appendix S2.** Inference of interspecific diet overlap using stable-isotope analysis between the two plover species, *Charadrius alexandrinus* and *C. dealbatus*. (DOCX 530 kb)

## Data Availability

Sequences deposited at GenBank: accession number MK830738- MK830957. Morphometric and geo-referred occurrence data, stable isotope profiles and microsatellite genotypes available at: Dryad Doi: doi:10.5061/dryad.3r01b8d

## Abbreviations

AMOVAs: Analyses of molecular variance
ANCOVA: Analyses of covariance
CR: The mitochondrial control region
*cytb*: Cytochrome b
ENM: Ecological niche modeling
*h*: Haplotype number
*Hd*: Haplotype
*H*_E_: Expected heterozygosity diversity
*H*o: Observed heterozygosity
IM: Isolation with Migration model
LD: Linkage disequilibrium
LGM: Last Glacial Maximum
LIG: Last Interglacial
MCMC: Markov chain Monte Carlo
mst: Microsatellites
mtDNA: Mitochondrial DNA
ND3: NADH dehydrogenase subunit 3 fragment
*N*e: Effective population size
PCA: Principal Component Analysis
*S*: Number of segregating sites
*π*: Nucleotide diversity
