# Supplementary material for: Genetic, phenotypic and ecological differentiation suggests incipient speciation in two Charadrius plovers along the Chinese coast
Source: BMC Evol Biol. 2019 Jun 27;19:135. doi: 10.1186/s12862-019-1449-5 (PMC6598359; doi:10.1186/s12862-019-1449-5)
Supplement: Supplementary file 1 — Table S1. Estimates of h, number of haplotypes; s, number of segregating sites; π, nucleotide diversity; Tajima’s D value, substitution rate of each locus estimated based on the substitution rate for cytb and GenBank accession number for each mtDNA and nuclear locus of C. alexandrinus and C. dealbatus were provided. The substitution rate of each locus was calculated using the method in Li et al. (2010): the ratio of net genetic distance of each locus across ingroup–outgroup was calculated, compared with net distance of mitochondrial cytochrome b (cytb) and then multiplied by the substitution rate for cytb (0.0105 ± 0.0005 substitution/site/mya, Weir & Schluter 2008). Table S2. Estimates of pairwise FST for each microsatellite locus between C. alexandrinus and C. dealbatus, LD test result (same for both species), Na, number of alleles, Ho, observed heterozygosity and He, expected heterozygosity in each species. Table S3. PCR amplification protocols for three mtDNA, 16 nuclear exons and 22 microsatellite loci genotyped for C. alexandrinus and C. dealbatus. Table S4. Genetic differentiation between each pair of sampling localities of C. alexandrinus and C. dealbatus. Estimates were based on 1729 bp mtDNA (lower diagonal, ΦST) and 13 microsatellite loci (upper diagonal, FST). Values highlighted in red represent significant value after Bonferroni correction. Table S5 The morphological measurements of C. alexandrinus and C. dealbatus in different breeding sites along the Chinese coast, and Taiwan and Hainan Islands. Site reference number corresponds to numbers in Fig. 1 and Table 1. The number of sample size for each site (n), the mean value of measurements including bill and wing length, body mass and their respective standard deviations (SD) are given. Only DNA samples from a breeding site in Cangzhou were collected but not the measurement, the corresponding data is missing for this population. Mean and SD of bill length, wing length and body mass for each population. M [file 12862_2019_1449_MOESM1_ESM.docx]

**Additional file 1**

**Genetic, phenotypic and ecological differentiation suggests incipient speciation in two *Charadrius* plovers along the Chinese coast**

Xuejing Wang^1,3§^, Pinjia Que^2§^, Gerald Heckel^3,5^, Junhua Hu^4^, Xuecong Zhang^1^, Chung-Yu Chiang^6^, Nan Zhang^1^, Qin Huang^1^, Simin Liu^1^, Jonathan Martinez^7^, Emilio Pagani-Núñez^1^, Caroline Dingle^8^, Leung Yu Yan^8^, Tamás Székely^1, 2, 9^, Zhengwang Zhang^2^, Yang Liu^1*^

1. State Key Laboratory of Biocontrol, Department of Ecology, School of Life Sciences, Sun Yat-sen University, Guangzhou 510275, China
2. Ministry of Education Key Laboratory for Biodiversity and Ecological Engineering, College of Life Sciences, Beijing Normal University, Beijing, 100875, China
3. Institute of Ecology and Evolution, University of Bern, Baltzerstrasse 6, 3012 Bern, Switzerland
4. Chengdu Institute of Biology, Chinese Academy of Sciences, Chengdu 610041, China
5. Swiss Institute of Bioinformatics, Genopode, 1015 Lausanne, Switzerland
6. Department of Environmental Science, Tunhai University, Taichun, Taiwan, China
7. 14, bis rue des Temples 45240 La Ferté Saint Aubin, France
8. School of Biological Sciences, The University of Hong Kong, Hong Kong S.A.R., China
9. Milner Center for Evolution, Department of Biology and Biochemistry, University of Bath, Bath BA1 7AY, UK

**Running title:** Speciation in *Charadrius* plovers

***Correspondence:** Yang Liu, School of Life Sciences, Sun Yat-sen University, Guangzhou, China. Email: [liuy353@mail.sysu.edu.cn](mailto:liuy353@mail.sysu.edu.cn)

**^§^** These authors contributed equally to this work.

**Table S1** Estimates of *h*, number of haplotypes; *s*, number of segregating sites; *π*, nucleotide diversity; *Tajima's D* value, substitution rate of each locus estimated based on the substitution rate for *cytb* and GenBank accession number for each mtDNA and nuclear locus of *C. alexandrinus* and *C. dealbatus* were provided. The substitution rate of each locus was calculated using the method in Li et al*.* (2010): the ratio of net genetic distance of each locus across ingroup–outgroup was calculated, compared with net distance of mitochondrial cytochrome b (*cytb*) and then multiplied by the substitution rate for *cytb* (0.0105 ± 0.0005 substitution/site/mya, Weir & Schluter 2008).

| **Locus** | **Length (bp)** | **n** | ***H*** | ***s*** | ***π*** | ***Tajima's D*** | **Substitution rate (x10^-8^)** | **GenBank Entries** |
| --- | --- | --- | --- | --- | --- | --- | --- | --- |
| ATPase6/8 | 846 | 357 | 16 | 15 | 0.00139 | -1.187 | - | MK830738-MK830753 |
| D-loop | 505 | 357 | 51 | 34 | 0.00360 | -1.856* | - | MK830765-MK830815 |
| ND3 | 378 | 357 | 11 | 10 | 0.00198 | -1.132 | - | MK830754-MK830764 |
| **mtDNA** | 1729 | 357 | 109 | 59 | 0.00216 | -1.741 | 27.389 | - |
| NFIL3 | 723 | 55 | 7 | 5 | 0.00051 | 0.554 | 1.191 | MK830949-MK830955 |
| SIPR3 | 718 | 64 | 2 | 1 | 0.00055 | 1.105 | 0.798 | MK830956-MK830957 |
| FMN2 | 679 | 80 | 28 | 18 | 0.00437 | -0.671 | 4.742 | MK830816-MK830843 |
| REST | 627 | 80 | 3 | 2 | 0.00046 | -0.455 | 1.129 | MK830844-MK830846 |
| MAML3 | 706 | 80 | 13 | 10 | 0.00170 | -1.073 | 1.483 | MK830847-MK830859 |
| TLR3 | 859 | 80 | 14 | 7 | 0.00252 | 1.296 | 1.797 | MK830860-MK830873 |
| INO80D | 902 | 80 | 8 | 8 | 0.00064 | -1.764 | 0.224 | MK830874-MK830881 |
| CCDC141 | 746 | 80 | 10 | 7 | 0.00139 | -0.645 | 1.587 | MK830882-MK830891 |
| CHST8 | 693 | 80 | 7 | 5 | 0.00118 | -0.428 | 2.445 | MK830892-MK830898 |
| NCOA6 | 750 | 80 | 12 | 7 | 0.00340 | 1.960 | 1.611 | MK830899-MK830910 |
| ADNP | 898 | 80 | 7 | 5 | 0.00051 | -1.217 | 0.942 | MK830911-MK830917 |
| LACTBL1 | 704 | 80 | 3 | 2 | 0.00029 | -0.782 | 1.354 | MK830918-MK830920 |
| HERC1 | 757 | 80 | 3 | 2 | 0.00048 | -0.159 | 1.140 | MK830921-MK830923 |
| BIRC2 | 692 | 80 | 20 | 14 | 0.00511 | 0.270 | 1.493 | MK830924-MK830942 |
| TMEM132B | 707 | 80 | 3 | 2 | 0.00007 | -1.408 | 0.673 | MK830943-MK830945 |
| USP38 | 741 | 80 | 3 | 2 | 0.00050 | -0.127 | 1.710 | MK830946-MK830948 |

**p* < 0.05; ***p* < 0.001.

**Table S2** Estimates of pairwise *F*_ST_ for each microsatellite locus between *C. alexandrinus* and *C. dealbatus*, LD test result (same for both species), *Na*, number of alleles, *Ho*, observed heterozygosity and *He*, expected heterozygosity in each species.

|  |  |  | ***C. alexandrinus*** | | | ***C. dealbatus*** | | |
| --- | --- | --- | --- | --- | --- | --- | --- | --- |
| **locus** | ***F*_ST_** | ***LD test*** | ***Na*** | ***Ho*** | ***He*** | ***Na*** | ***Ho*** | ***He*** |
| Calex-01 | 0.006 | - | 13 | 0.720 | 0.738 | 10 | 0.622 | 0.656 |
| Calex-02 | 0.006* | - | 18 | 0.833 | 0.834 | 12 | 0.865 | 0.834 |
| Calex-05 | 0.008* | - | 8 | 0.683 | 0.717 | 6 | 0.609 | 0.637 |
| Calex-12 | 0.006 | - | 9 | 0.516 | 0.781 | 9 | 0.654 | 0.757 |
| Calex-14 | 0.006* | C204 | 23 | 0.772 | 0.892 | 16 | 0.891 | 0.873 |
| Calex-18 | 0.011* | - | 12 | 0.748 | 0.809 | 11 | 0.833 | 0.825 |
| Calex-22 | 0.043* | - | 8 | 0.614 | 0.675 | 4 | 0.519 | 0.582 |
| Calex-23 | 0.002 | - | 21 | 0.740 | 0.813 | 16 | 0.603 | 0.743 |
| Calex-32 | 0.006 | - | 14 | 0.695 | 0.731 | 10 | 0.744 | 0.752 |
| Calex-35 | 0.014* | - | 29 | 0.679* | 0.891 | 23 | 0.667* | 0.820 |
| Calex-37 | 0.003 | - | 22 | 0.634 | 0.889 | 20 | 0.808 | 0.892 |
| Calex-39 | 0.011* | - | 33 | 0.866 | 0.916 | 26 | 0.865 | 0.888 |
| Calex-45 | 0.010* | - | 16 | 0.809 | 0.881 | 16 | 0.774 | 0.878 |
| C204 | 0.012* | Calex-14 | 32 | 0.874 | 0.925 | 18 | 0.872 | 0.877 |
| Hru2 | 0.002 | - | 7 | 0.667 | 0.722 | 9 | 0.679 | 0.756 |

**p* < 0.05.

**Table S3** PCR amplification protocols for three mtDNA, 16 nuclear exons and 22 microsatellite loci genotyped for *C. alexandrinus* and *C. dealbatus.*

|  |  | **Initial denaturation** | **Denaturation** | **Annealing** | **Extension** | **cycles** | **final extension** |
| --- | --- | --- | --- | --- | --- | --- | --- |
| **ATPase6/8** | Temp/°C | 94 | 94 | 60 | 72 | 35 | 72 |
|  | Duration | 5min | 30s | 30s | 60s |  | 8min |
| **D-loop and ND3** | Temp/°C | 94 | 94 | 50 | 72 | 35 | 72 |
|  | Duration | 5min | 30s | 30s | 60s |  | 8min |
| **Nuclear loci** | Temp/°C | 94 | 94 | 62 to 52 in 10 cycles, followed by 52 for 30 cycles | 72 | 10+30 | 72 |
|  | Duration | 2min | 30s | 30s | 60s |  | 8min |
| **Microsatellites** | Temp/°C | 95 | 94 | 57/60/62 | 72 | 35 | 72 |
|  | Duration | 15min | 30s | 90s | 90s |  | 10min |
|  | Multi 57°C | Calex-02, 04, 05, 08, 18, 19, 23, 24,39, 43, 45 | | | | | |
|  | Multi 60°C | Calex-32, 35; C204; Hru2 | | | | | |
|  | Multi 62°C | Calex-01, 11, 12, 14, 26, 31,37 | | | | | |

**Table S4** Genetic differentiation between each pair of sampling localities of *C. alexandrinus* and *C. dealbatus*. Estimates were based on 1729 bp mtDNA (lower diagonal, *Φ*_ST_) and 13 microsatellite loci (upper diagonal, *F*_ST_). Values highlighted in red represent significant value after Bonferroni correction.

| **Qinghai Lake** | **Tangshan** | **Cangzhou** | **Weifang** | **Lianyungang** | **Rudong** | **Zhoushan** | **Ningbo** | **Wenzhou** | **Xinbei** | **Zhanghua** | **Jinmen** | **Fuzhou** | **Xiamen** | **Shanwei** | **Xitou** | **Zhanjiang** | **Beihai** | **Dongfang** | **mst mtDNA** |
| --- | --- | --- | --- | --- | --- | --- | --- | --- | --- | --- | --- | --- | --- | --- | --- | --- | --- | --- | --- |
|  | 0.062 | 0.012 | 0.008 | 0.009 | 0.013 | -0.002 | 0.009 | 0.033 | 0.041 | 0.033 | 0.044 | 0.062 | 0.060 | 0.021 | 0.012 | 0.004 | 0.007 | 0.008 | **Qinghai Lake** |
| 0.085 |  | 0.032 | 0.059 | 0.073 | 0.059 | 0.055 | 0.040 | 0.034 | 0.039 | 0.032 | 0.039 | 0.037 | 0.036 | 0.030 | 0.072 | 0.054 | 0.066 | 0.041 | **Tangshan** |
| 0.711 | 0.597 |  | 0.008 | 0.015 | 0.020 | 0.010 | 0.007 | 0.011 | 0.011 | 0.015 | 0.031 | 0.040 | 0.029 | 0.015 | 0.021 | 0.011 | 0.013 | 0.006 | **Cangzhou** |
| -0.004 | 0.103 | 0.773 |  | 0.007 | 0.011 | 0.006 | 0.006 | 0.043 | 0.047 | 0.035 | 0.055 | 0.060 | 0.057 | 0.028 | 0.018 | 0.017 | 0.011 | 0.012 | **Weifang** |
| 0.002 | 0.070 | 0.586 | 0.041 |  | 0.009 | 0.011 | 0.012 | 0.043 | 0.055 | 0.046 | 0.065 | 0.086 | 0.076 | 0.038 | 0.024 | 0.012 | 0.010 | 0.014 | **Lianyungang** |
| 0.519 | 0.485 | -0.004 | 0.551 | 0.433 |  | 0.013 | 0.012 | 0.033 | 0.052 | 0.046 | 0.059 | 0.070 | 0.065 | 0.030 | 0.029 | 0.019 | 0.013 | 0.018 | **Rudong** |
| 0.549 | 0.499 | -0.006 | 0.583 | 0.456 | 0.007 |  | 0.007 | 0.039 | 0.043 | 0.030 | 0.044 | 0.059 | 0.055 | 0.017 | 0.011 | 0.001 | 0.003 | 0.005 | **Zhoushan** |
| 0.661 | 0.601 | 0.006 | 0.698 | 0.580 | 0.029 | 0.068 |  | 0.032 | 0.034 | 0.033 | 0.049 | 0.060 | 0.053 | 0.019 | 0.027 | 0.010 | 0.011 | 0.004 | **Ningbo** |
| 0.532 | 0.493 | 0.012 | 0.564 | 0.437 | 0.006 | 0.021 | 0.066 |  | -0.007 | 0.016 | 0.015 | 0.031 | 0.040 | 0.025 | 0.057 | 0.029 | 0.035 | 0.022 | **Wenzhou** |
| 0.092 | 0.145 | 0.749 | 0.122 | 0.092 | 0.558 | 0.588 | 0.695 | 0.570 |  | 0.019 | 0.018 | 0.031 | 0.039 | 0.019 | 0.057 | 0.038 | 0.040 | 0.026 | **Xinbei** |
| 0.738 | 0.699 | 0.519 | 0.751 | 0.673 | 0.457 | 0.472 | 0.540 | 0.488 | 0.750 |  | 0.012 | 0.021 | 0.020 | 0.017 | 0.043 | 0.030 | 0.043 | 0.018 | **Zhanghua** |
| 0.588 | 0.530 | -0.033 | 0.627 | 0.486 | -0.012 | -0.002 | 0.046 | 0.017 | 0.626 | 0.464 |  | 0.017 | 0.026 | 0.029 | 0.064 | 0.040 | 0.052 | 0.039 | **Jinmen** |
| 0.563 | 0.505 | 0.045 | 0.612 | 0.437 | 0.002 | 0.043 | 0.052 | 0.034 | 0.608 | 0.502 | 0.020 |  | -0.002 | 0.021 | 0.068 | 0.054 | 0.063 | 0.047 | **Fuzhou** |
| 0.652 | 0.553 | 0.017 | 0.718 | 0.519 | -0.005 | 0.041 | 0.082 | 0.041 | 0.696 | 0.520 | -0.026 | 0.014 |  | 0.021 | 0.058 | 0.052 | 0.066 | 0.037 | **Xiamen** |
| 0.039 | 0.086 | 0.669 | 0.061 | 0.006 | 0.508 | 0.535 | 0.641 | 0.518 | 0.117 | 0.720 | 0.567 | 0.535 | 0.611 |  | 0.015 | 0.006 | 0.023 | 0.010 | **Shanwei** |
| 0.143 | 0.028 | 0.451 | 0.212 | 0.068 | 0.309 | 0.341 | 0.456 | 0.322 | 0.228 | 0.609 | 0.373 | 0.310 | 0.398 | 0.127 |  | 0.008 | 0.018 | 0.017 | **Xitou** |
| 0.777 | 0.643 | 0.049 | 0.846 | 0.649 | 0.030 | 0.065 | 0.090 | 0.120 | 0.809 | 0.528 | 0.026 | 0.140 | 0.082 | 0.724 | 0.510 |  | -0.006 | 0.002 | **Zhanjiang** |
| -0.019 | 0.041 | 0.602 | 0.001 | -0.014 | 0.450 | 0.476 | 0.591 | 0.462 | 0.070 | 0.689 | 0.506 | 0.463 | 0.538 | 0.028 | 0.051 | 0.656 |  | 0.010 | **Beihai** |
| 0.678 | 0.589 | -0.005 | 0.726 | 0.571 | 0.007 | 0.049 | 0.000 | 0.041 | 0.714 | 0.532 | 0.028 | 0.023 | 0.064 | 0.646 | 0.429 | 0.097 | 0.587 |  | **Dongfang** |

**Table S5** The morphological measurements of *C. alexandrinus* and *C. dealbatus* in different breeding sites along the Chinese coast, and Taiwan and Hainan Islands. Site reference number corresponds to numbers in Figure 1 and Table 1. The number of sample size for each site (n), the mean value of measurements including bill and wing length, body mass and their respective standard deviations (*SD*) are given. Only DNA samples from a breeding site in Cangzhou were collected but not the measurement, the corresponding data is missing for this population. Mean and SD of bill length, wing length and body mass for each population. Measurement data of the site 3-Cangzhou was not collected.

| **Site** | | ***n*** | **Bill length** | | **Wing length** | | **Body mass** | |
| --- | --- | --- | --- | --- | --- | --- | --- | --- |
|  |  |  | **Mean/mm** | ***SD*** | **Mean/mm** | ***SD*** | **Mean/g** | ***SD*** |
| 1 | Qinghai Lake | 62 | 15.72 | 0.50 | 117.39 | 9.62 | 46.34 | 9.25 |
| 2 | Tangshan | 80 | 16.56 | 0.65 | 115.85 | 7.27 | 47.33 | 9.67 |
| 3 | Cangzhou | 0 | - | - | - | - | - | - |
| 4 | Weifang | 16 | 17.30 | 0.82 | 117.00 | 9.87 | 48.30 | 10.09 |
| 5 | Lianyungang | 11 | 16.35 | 0.20 | 113.73 | 7.42 | 45.36 | 12.94 |
| 6 | Rudong | 12 | 16.95 | 0.47 | 115.08 | 3.54 | 45.53 | 6.90 |
| 7 | Zhoushan | 8 | 16.73 | 0.64 | 116.25 | 3.36 | 43.72 | 5.48 |
| 8 | Ningbo | 11 | 16.84 | 0.10 | 114.73 | 4.22 | 43.95 | 7.30 |
| 9 | Wenzhou | 66 | 16.86 | 0.54 | 113.57 | 6.39 | 48.86 | 10.93 |
| 10 | Xinbei | 14 | 18.73 | 0.44 | 115.43 | 8.61 | 52.71 | 28.61 |
| 11 | Zhanghua | 11 | 18.62 | 0.87 | 116.73 | 6.07 | 51.37 | 4.63 |
| 12 | Fuzhou | 27 | 18.74 | 0.67 | 119.67 | 9.23 | 51.39 | 6.15 |
| 13 | Xiamen | 22 | 17.60 | 0.77 | 119.27 | 6.02 | 48.93 | 5.68 |
| 14 | Jinmen | 15 | 18.49 | 0.28 | 115.73 | 50.03 | 50.43 | 12.07 |
| 15 | Shanwei | 25 | 17.53 | 0.63 | 118.12 | 4.94 | 48.05 | 5.51 |
| 16 | Xitou | 3 | 17.78 | 0.64 | 111.00 | 3.00 | 45.99 | 83.65 |
| 17 | Zhanjiang | 14 | 17.43 | 0.52 | 118.79 | 7.41 | 46.20 | 7.05 |
| 18 | Beihai | 20 | 17.50 | 0.48 | 117.75 | 5.78 | 47.92 | 14.50 |
| 19 | Dongfang | 2 | 17.27 | 1.05 | 120.50 | 4.50 | 42.81 | 2.02 |

**Table S6** Pearson’s correlation coefficients between pairwise of bioclimatic variables with the ranges of *C. alexandrinus* and *C. dealbatus*.

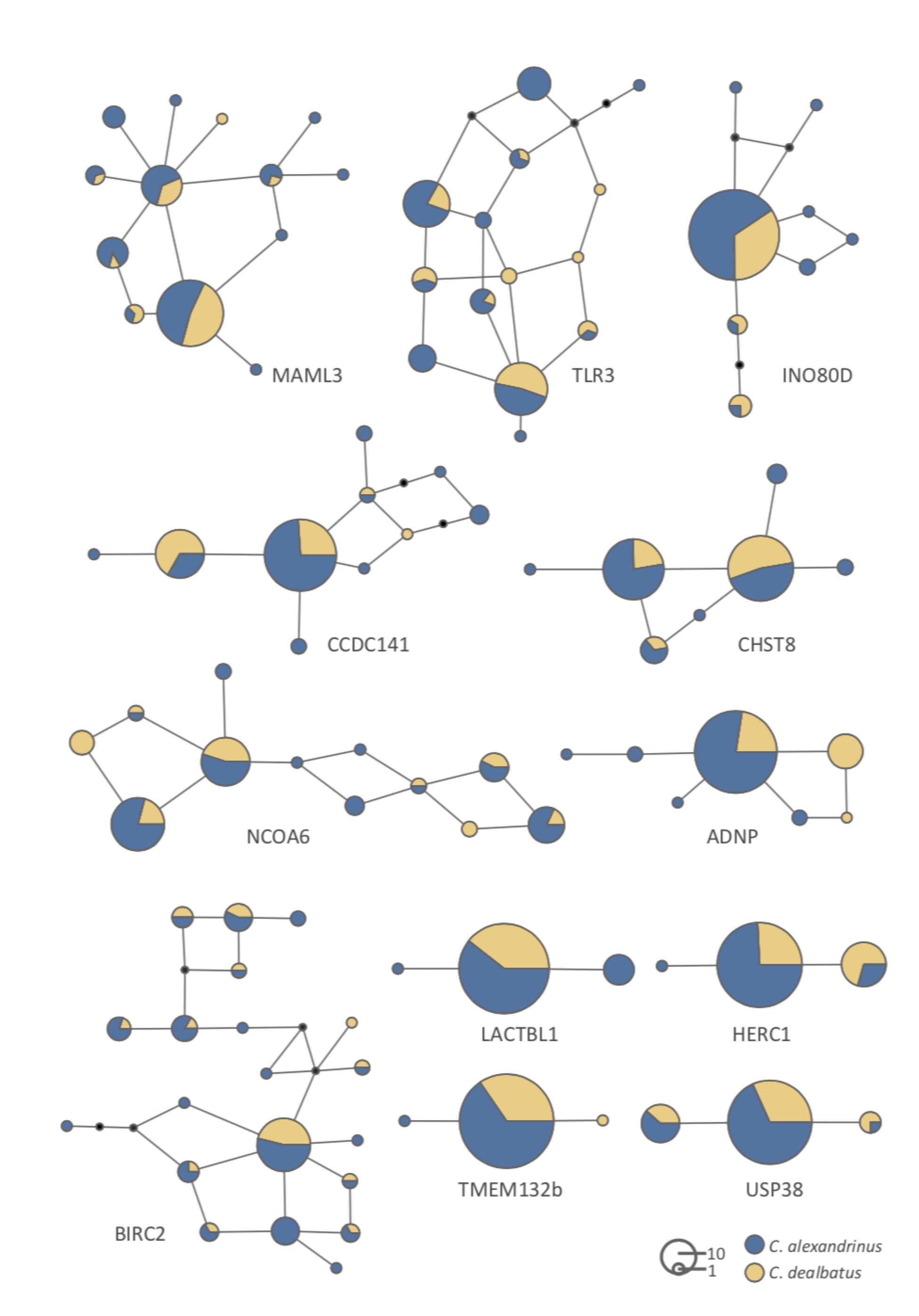


**Figure S1** Haplotype networks based on 80 individuals of 12 nuclear loci not shown in Figure 2. *C. alexandrinus* (blue) and *C. dealbatus* (yellow).


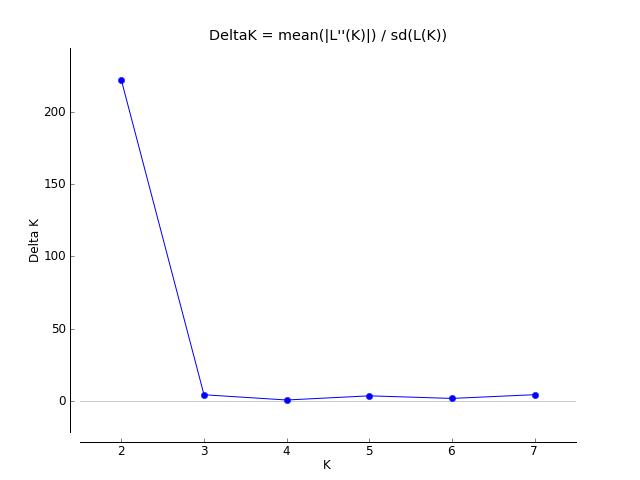


**Figure S2** The Bayesian clustering analysis with STRUCTURE clearly suggested two genetic clusters corresponding to *C. alexandrinus* and *C. dealbatus*. Shown is the maximum value of the Delta K (**Δ** K) in posterior likelihood Ln P (X/K) over 10 runs per K of STRUCTURE.


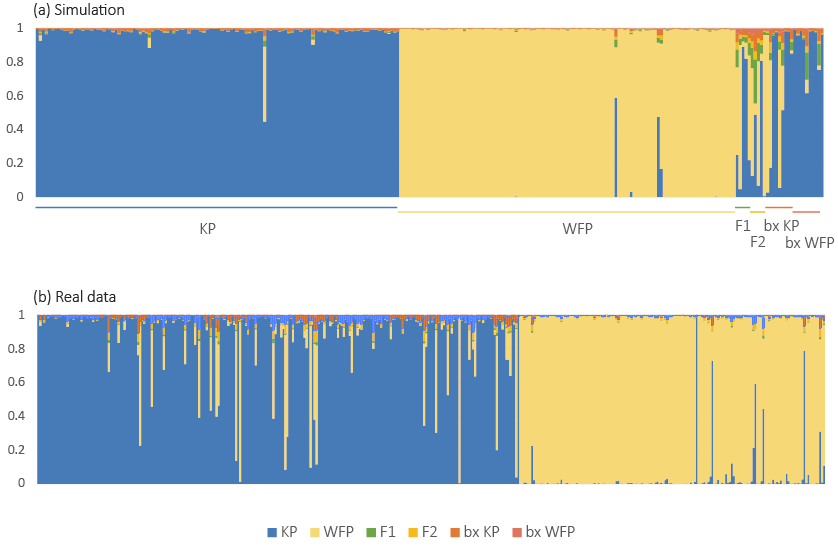


**Figure S3** (a) Newhybrid simulations failed to reliably detect simulated hybrid individuals. Real data of individuals with posterior density higher than 95% in STRUCTURE, and data of 5 simulated F1 and F2 individuals, and 10 back-cross individuals on each direction was used to imitate a situation when hybrids were the minority in the population. (b) Newhybrid results from real data are highly consistent with the STRUCTURE results. KP represents *C. alexandrinus*, WFP represents *C. dealbatus*, bx represents back-crosses.


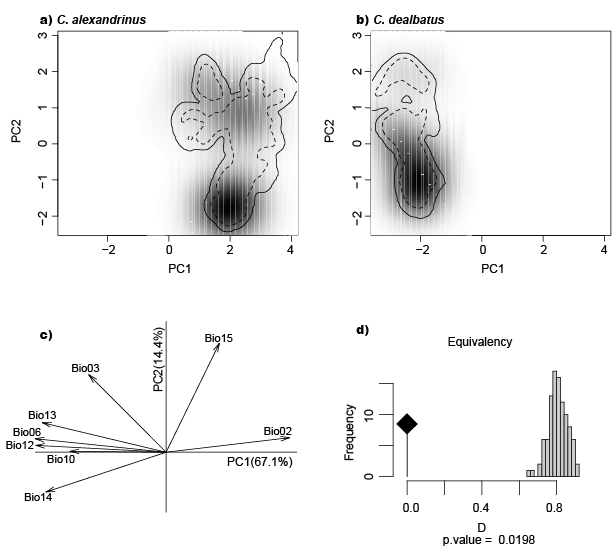


**Figure S4** Niche of *C. alexandrinus* and *C. dealbatus* in climatic space from a principal component analysis (PCA-env). a) and b) show the niche characteristics of *C. alexandrinus* and *C. dealbatus*, respectively, along the first two axes of the PCA. Grey shading shows the density of the occurrences of the species by cell. The solid and dashed contour lines illustrate, respectively, 100% and 50% of the available (background) environment. c) The contribution of the variables on the first two axes of the PCA and the percentage of inertia explained by the two axes. d) Observed niche overlap D between the two ranges (bars with a diamond) and simulated niche overlaps (grey bars) on which tests of niche equivalency were calculated with 100 iterations.


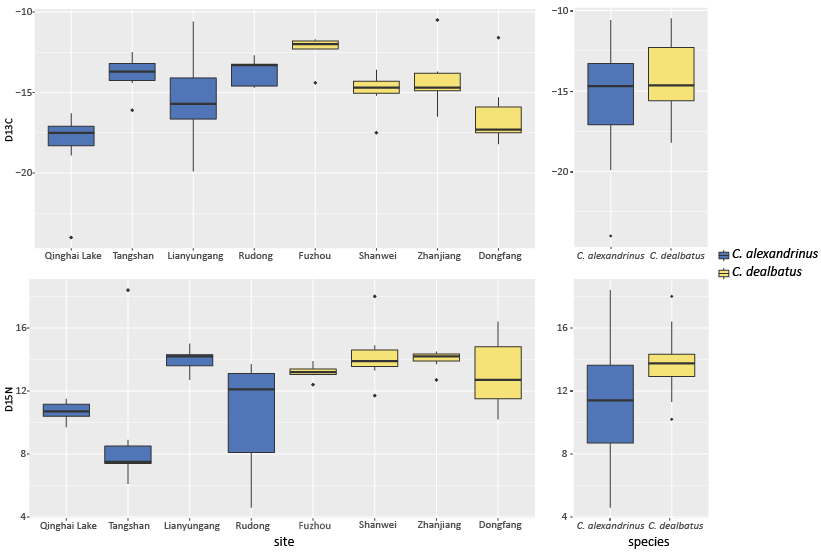


**Figure S5** Differentiation in the stable isotope ratios among breeding sites (top-left: δ^13^C and bottom-left: δ^15^N) and between the two plovers (top-right: δ^13^C and bottom-right: δ^15^N). In all representation, *C. alexandrinus* marked in blue and *C. dealbatus* in yellow.

**Appendix S1.** **Reconstruction of potential range shifts induced by climatic changes and inference of environmental niche overlap between the two-plover species, *Charadrius alexandrinus* and *C. dealbatus*.**

To infer potential range shifts induced by climatic changes, we carried out Ecological Niche Modeling (ENM) using Maxent 3.3.3k (Phillips et al. 2006; Phillips and Dudik, 2008). The occurrence records of the two-plover species were obtained from multiple sources including our field surveys between 2002-2014 year, and the citizen science database “China Bird Report” (http://www.birdreport.cn/). We treated all occurrences equally regardless of the size of populations, and mapped all records using ArcGIS 9.2 (ESRI, Redland, CA) for visual inspection to detect duplicates and possible errors in georeferencing. We filtered the unique georeferenced records using a 0.1°-resolution grid and included a single randomly selected occurrence per grid-cell to reduce the influence of sample selection bias (Phillips et al. 2009) and spatial autocorrelation. A final compilation of 93 occurrence records for KP, and 24 for WEP was attained. We derived 19 bioclimatic variables representing climatic conditions (for the period 1960-1990, the last glacial maximum (LGM, ca. 21,000 year ago (21 kyr BP)) and interglacial periods (LIG, ca. 120-100 kyr BP) within the study area with a resolution of 0.1 arc-degrees from the WorldClim database v.1.4 (Hijmans et al. 2005) and retained the variables that gave a higher value in the regularized gain to the Maxent model (Phillips et al., 2006) for highly correlated variable pairs (|r| ≥ 0.8) (Hu & Jiang 2010). Consequently, eight variables were used, including the mean diurnal range, isothermality, minimum temperature of the coldest month, mean temperature of the warmest quarter, annual precipitation, precipitation of the wettest month, precipitation of the driest month and precipitation seasonality. We mainly employed default settings and ran models with ten cross-validate replicates. We assessed discrimination abilities of the ENMs using the area under the receiver operating characteristic curve (AUC) (Swets 1988), and calculated the average value of replicates. The logistic output format was used with suitability values ranging from 0 (lowest) to 1 (highest) (Phillips & Dudík 2008). The mean suitability of replicate outputs was calculated within each grid-cell for further processing in ArcGIS 9.2 (ESRI, Redland, CA). Moreover, we projected the paleo-distributions based on current ENMs. When projecting paleo-distributions, we used the selected environmental variables as used for current conditions. Models were projected to environmental variables in the Last Glacial Maximum (LGM; 21 000 years ago) and interglacial periods (LIG, c.a 120-100 kyr BP). Both the community climate system model (CCSM) (Collins et al. 2006) and model for interdisciplinary research on climate (MIROC) (Hasumi & Emori 2004) were used to generate predictions for distributions during the LGM.

To explore niche comparison between the two species, we performed an ordination null test of PCA-env in environmental (E)-space (Broennimann et al. 2012). The PCA-env can most accurately retrieve the simulated level of niche overlap among ordination techniques without substantial bias (Broennimann et al. 2012). An unbiased estimate of Schoener’s D metric was calculated for our data using smoothed densities from a kernel density function to measure niche overlap between the two species that is ensured to be independent of the resolution of the grid. Statistical confidence in niche overlap values was then tested through a one-sided niche-similarity test (Broennimann et al. 2012). The observed overlap values greater than the simulated values indicate that niches of the clade pair under comparison are more similar than random. We delimited the background using the prediction of each species’ threshold inferred from ENMs (Hu et al., 2016). All statistical analyses were performed in R 3.0.2 (R Development Core Team 2013) using scripts available in Broennimann et al. (2012).

**Appendix S2** **Inference of interspecific diet overlap using stable-isotope analysis between the two plover species, *Charadrius alexandrinus* and *C. dealbatus*.**

We collected the outer pair of rectrices from seven adults per site at overall eight sites: Qinghai Lake, Tangshan, Lianyungang and Rudong for *C. alexandrinus*; Fuzhou, Shanwei, Zhanjiang, Dongfang for *C. dealbatus*. Since both species perform a complete post-breeding molt within their breeding grounds (Ginn & Melville 1983, personal observation), isotope ratios represent trophic level and habitat preference during the breeding period.

Feathers were washed with a 2:1 chloroform-methanol solution, and then with deionized water. After drying in air, we cut the vein of each feather sample into pieces, loaded about 0.5 mg in tin recipients, and burned for combustion. According to a protocol described in Pagani-Núñez et al. (2017), we determined δ^15^N and δ^13^C in terms of the following formula: δX=[(R_sam_-R_std_)/R_std_] x 1000=(R_sam_/R_std_-1) x 1000, in which R_sam_ means the element abundance of the sample, and R_std_ means the element abundance of the international standard (e.g. Pagani-Núñez et al. 2017). We carried out isotopic analysis in the Stable Isotope Ratio Mass Spectrometry (SIRMS) laboratory at the University of Hong Kong, via combustion in a Eurovector EA3028 coupled to a Perspective IRMS (Nu Instrument). Analytical precision was determined by repeated analysis of an internal acetanilide standard ('acet 6'; 70%C). Mean (± SE) precision during analysis was 0.2 ± 0.04 and 0.1 ± 0.01 for δ^13^C and δ^15^N, respectively.

We used the packages “ggplot2” (Wickham, 2009) and “nicheROVER” (Swanson et al. 2015) in R v3.3.1 (R Core Team, 2015) to compute isotope niche regions and interspecific niche overlap. We displayed 10 random niche regions generated by the Bayesian analysis, and calculated overlap metric estimates from 1,000 Monte Carlo simulations with a probability level of alpha=0.95. We calculated niche overlap as the probability to find an individual of a species A in the niche region of the other compared species; and generated the figures as shown by elliptical projections based on 10 random niche regions (Swanson *et al.* 2015).

**References**

Broennimann, O., Fitzpatrick, M.C., Pearman, P.B., Petitpierre, B., Pellissier, L., Yoccoz, N.G., Thuiller, W., Fortin, M.-J., Randin, C., Zimmermann, N.E., Graham, C.H. & Guisan, A. (2012) Measuring ecological niche overlap from occurrence and spatial environmental data. *Global Ecology and Biogeography* **21,** 481-497.

Collins, W.D., Bitz, C.M., Blackmon, M.L., Bonan, G.B., Bretherton, C.S., Carton, J.A., Chang, P., Doney, S.C., Hack, J.J., Henderson, T.B., Kiehl, J.T., Large, W.G., McKenna, D.S., Santer, B.D. & Smith, R.D. (2006) The Community Climate System Model Version 3 (CCSM3). *Journal of Climate* **19**, 2122-2143.

Ginn, H. & Melville, D. (1983) Moult in birds. BTO Guide 19. British Trust for Ornithology, Tring, UK.

Hasumi, H. & Emori, S. (2004) K-1 coupled GCM (MIROC) Description. In: <http://www>. ccsr. u-tokyo. ac. jp/kyosei/hasumi/MIROC/tech-repo. pdf (ed. U.O.T. Center for Climate System Research), Tokyo.

Hijmans, R.J., Cameron, S.E., Parra, J.L., Jones, P.G. & Jarvis, A. (2005) Very high resolution interpolated climate surfaces for global land areas. *International Journal of Climatology* **25**, 1965-1978.

Hu, J., Broennimann, O., Guisan, A., Wang, B., Huang, Y., Jiang, J. (2016) Niche conservatism in Gynandropaa frogs on the southeastern Qinghai-Tibetan Plateau. *Scientific Reports* **6**, 32624.

Pagani-Núñez, E., Renom, M., Mateos-Gonzalez, F., Cotín, J. and Senar, J.C., (2017). The diet of great tit nestlings: Comparing observation records and stable isotope analyses. *Basic and Applied Ecology* **18**, 57-66.

Phillips, S.J., Dudik, M., Elith, J., Graham, C.H., Lehmann, A., Leathwick, J. & Ferrier, S. (2009) Sample selection bias and presence-only distribution models: implications for background and pseudo-absence data. *Ecological Applications* **19**, 181-197.

R Development Core Team (2013) R: a language and environment for statistical computing. Available at: <http://www.rproject.org/>

Swanson, H.K., Lysy, M., Power, M., Stasko, A.D., Johnson, J.D. & Reist, J.D. (2015) A new probabilistic method for quantifying n‐dimensional ecological niches and niche overlap. *Ecology* **96**, 318-324.

Swets, J.A. (1988) Measuring the accuracy of diagnostic systems. *Science* **240**, 1285-1293.
